# Differences in metabolic adaptations during mid and late pregnancy: a comparative cohort study between Rwanda and Germany

**DOI:** 10.7189/jogh.15.04206

**Published:** 2025-07-11

**Authors:** Alemayehu Amberbir, Madeleine Ordnung, Sage Marie Consolatrice Ishimwe, Ronald Biemann, Mandy Vogel, Wieland Kiess, Antje Körner, Balkachew Nigatu, Darius Bazimya, Theogene Uwizeyimana, Jean Baptiste Niyibizi, Daniel Seifu, Abebe Bekele, Jon Genuneit

**Affiliations:** 1 Center for Population Health, University of Global Health Equity, Kigali, Rwanda; 2 Pediatric Epidemiology, Department of Pediatrics, Leipzig University, Leipzig, Germany; 3 Institute of Laboratory Medicine, Clinical Chemistry and Molecular Diagnostics, University Hospital Leipzig, Leipzig, Germany; 4 LIFE Leipzig Research Center for Civilization Diseases, University of Leipzig, Leipzig, Germany; 5 Department of Women and Child Health, University of Leipzig, Hospital for Children and Adolescents and Center for Pediatric Research, Leipzig, Germany; 6 King Faisal Hospital, Kigali, Rwanda; 7 Bill and Joyce Cummings Institute of Global Health, University of Global Health Equity, Kigali, Rwanda; 8 School of Medicine, University of Global Health Equity, Butaro, Rwanda; 9 School of Medicine, University of Global Health Equity, Kigali, Rwanda; 10 German Center for Child and Adolescent Health partner site Leipzig-Dresden, Leipzig, Germany

## Abstract

**Background:**

While cross-ancestral differences in glucose and lipid metabolism are widely reported in adults, there is a paucity of data on pregnant women during various stages of pregnancy. There is no consensus on what defines normal lipid ranges during pregnancy. Establishing reference ranges is crucial to reduce the risk of missing associated maternal and fetal health issues. Therefore, we aimed to investigate the metabolic profiles of healthy pregnant women and to establish national Rwandan reference ranges for these metabolites.

**Methods:**

We derived the data from two ongoing longitudinal cohort studies conducted in predominantly rural Rwanda and urban Germany (Leipzig), providing repeat data from the second and third trimesters of pregnancy. We measured concentrations of glucose, total cholesterol (TC), high-density lipoprotein cholesterol (HDL), and triglycerides (TG), and estimated their associations with trimesters and cohorts using multivariable linear regression. We estimated the reference ranges using the 5th and 95th percentiles for each metabolic marker.

**Results:**

For Rwanda and Leipzig, lipids and lipoproteins increased across trimesters, except for HDL, which remained equally low for Rwanda and significantly decreased for Leipzig. Concentrations of TC, low-density lipoprotein, and non-HDL were significantly higher in Leipzig compared to Rwanda for both trimesters, while HDL was significantly lower in Rwanda. Rwanda exhibited significantly higher TG levels in the second trimester than Leipzig, although this difference did not persist into the third trimester. Glucose concentrations were significantly higher in Rwanda than in Leipzig for both trimesters.

**Conclusions:**

This is the first representative study investigating lipid and lipoprotein concentrations in pregnant women from Rwanda and comparing them to a European sample. This study shows that lipid, lipoprotein, and glucose concentrations differ by ancestry and stage of pregnancy. The higher TG and glucose concentrations in Rwanda may indicate an emerging burden of metabolic disorders in Africa.

Metabolic adaptations during pregnancy are essential for pregnancy maintenance and optimal fetal development. Substantial changes in glucose and lipid metabolism ensure maximum nutrient availability for fetal growth [1]. As the fetus depends on maternal glucose as the primary energy substrate, glucose homeostasis is maintained through compensatory changes in insulin secretion, insulin action and increases in hepatic glucose production [2]. In conjunction with lipolysis of additional maternal adipose tissue accumulated during early pregnancy, circulating concentrations of cholesterol and triglycerides (TG) steadily increase throughout pregnancy [3].

Literature shows that unfavourable metabolic profiles during different stages of pregnancy are associated with several adverse maternal and fetal outcomes. For instance, elevated or decreased concentrations of TG, total cholesterol (TC), low-density lipoprotein (LDL) and high-density lipoprotein cholesterol (HDL) are associated with preterm birth [4,5], being small- or large-for-gestational age (GA) [4,6], pre-eclampsia [5,7,8] and gestational diabetes mellitus (GDM) [5]. Hyperglycemia, even below those diagnostics of diabetes, has been shown to increase the risk of GDM [9], elevated birth weight [10,11], and premature birth [11].

For the general population, studies consistently report varying lipid profiles by ancestry, with individuals of African origin displaying lower concentrations of TG and higher levels of HDL [12]. However, only a few studies investigated ancestral differences in lipid and glucose metabolism in pregnant women. These studies indicate that compared with Caucasian pregnant women, women of African origin show decreased levels of TC and TG [13–15] and elevated concentrations of HDL [13,14] during early and mid-gestation, as well as smaller increases in TG, TC, and LDL [16] during the second trimester. Fasting glucose levels are lower in pregnant women of African origin than in Caucasians [17,18].

Furthermore, there is currently no consensus on what defines normal lipid ranges during pregnancy [19]. Thus, previously shown ancestral differences in lipid profiles highlight the need for national reference ranges to prevent the potential risk of overlooking associated maternal and fetal health issues. Although studies have attempted to establish reference ranges for Caucasian [20,21] and Asian [22,23] populations, reference ranges for African populations are still lacking.

We aimed to investigate the metabolic profiles and their trajectories over gestation in healthy pregnant women of different ancestries and stages of pregnancy, using data from two large cohort studies conducted in a predominantly rural population in Rwanda (East Africa) and urban Germany (city of Leipzig). Additionally, we aimed to establish reference ranges for maternal lipids, lipoproteins, and glucose.

## METHODS

### Study population and design

Rwanda is a country located in East Africa with a population of about 13 million. This study is part of an ongoing birth cohort study with enrolment in the second trimester of pregnancy from January 2023 to June 2023. We included pregnant women receiving health care in selected health centres within three districts: Burera (Northern Province, with four health centres), Nyanza (Southern Province, with three health centres), and Bugesera (Eastern Province, with four health centres). Participation was voluntary, and we obtained written informed consent from all participants. We maintained confidentiality throughout the study. We collected a sample of 303 women from Rwanda, aged 18–47 years. We examined the women during the second and third trimesters of pregnancy.

Leipzig is a city and home to about 616 000 of the 84 million inhabitants of Germany. Leipzig Research Centre for Civilization Diseases (LIFE) Child is a panel study of children and adolescents recruited from the general population in Leipzig since 2011 as part of the LIFE [24]. The LIFE Child also includes the recruitment of pregnant women in the second trimester of pregnancy. A total of 623 women between the ages of 18 and 43 years were examined during the second and third trimester of pregnancy.

### Data collection and measures

In the Rwandan and Leipzig cohorts, we collected repeat data in the second and third trimesters of pregnancy. For both cohorts, we instructed the participants to fast for at least eight hours prior to blood withdrawal. However, we did not use non-compliance with fasting instructions as an exclusion criterion. Therefore, the primary analysis included all individuals regardless of their fasting status. We conducted a sensitivity analysis specifically for those who fasted.

In Rwanda, trained study staff collected the data on demographics, pregnancy, and medical history through face-to-face interviews. The trained study staff objectively measured anthropometrics (weight, height, and mid-upper arm circumference (MUAC)) according to a WHO standard operating procedure. We measured glucose, TC, HDL, and TG in capillary whole blood from a finger prick with point-of-care testing following the manufacturer’s protocols. We used a glucose oxidase strip method on Call® Extra Glucose Monitoring System (ACON Laboratories, Inc.) for glucose measurements. We measured TC, HDL, LDL, and TG using the Cobas b 101 system (Roche Diagnostics), which is intended for use in a clinical laboratory or point-of-care setting. The Cobas b 101 system determines TC, HDL, and TG by enzymatic colourimetric methods. We calculated LDL using the Friedewald formula for samples with TG<4.52 mmol/L. In samples with higher TG levels, LDL was not reported. The instrument also provided a calculated value for non-HDL. A medical doctor accompanied the data collectors in the study sites to provide counselling services and facilitate referral when needed.

In Leipzig, we measured metabolic markers at the Institute for Laboratory Medicine of the University Hospital using venous blood samples. We processed the blood samples for serum collection within 30 minutes. We performed glucose and serum lipid measurements on a Cobas 8000 automated laboratory analyser (Roche Diagnostics) according to the manufacturer's protocols. We used the hexokinase method for glucose measurement. We determined TC, HDL, LDL, and TG by specific enzymatic colourimetric methods. We calculated the non-HDL. We assessed demographics and medical history by standardised interviews and questionnaires, and obtained pregnancy-related information from paper-based routine documentation of pregnancy. Trained staff measured anthropometrics in a standardised way.

We calibrated and quality-checked all devices for laboratory measurements as per the manufacturer’s instructions.

### Data analysis

We descriptively analysed and visualised the data. We compared concentrations of TC, HDL, LDL, non-HDL, TG, and glucose across trimesters and between cohorts using the Mann-Whitney test for location and the Siegel-Tukey test for scale. We estimated the associations between trimester and cohort and the concentrations of the biomarkers using adjusted linear regression. We selected potential confounders based on data availability in both studies and their importance, as determined by a directed acyclic graph: maternal age, GA, gravidity, and MUAC. We designated the second trimester and the Rwandan cohort as the reference categories. We performed sensitivity analyses, restricting the cohorts to assessment at specific GAs or to individuals who reported fasting for at least eight hours. For these analyses, we used SAS, version 9.4 (The SAS Institute, Cary, North Carolina, USA), *R*, version 4.4 (R Core Team, Vienna, Austria), and the GAMLSS package, version 6.0-6.

We estimated reference ranges of both cohorts by using the 5th and 95th percentiles for each of the metabolic measurements. We modelled the distribution parameters using generalised additive models for location, shape, and scale, assuming a Box-Cox-power-exponential distribution (four-parameter distribution) [25]. After checking for nonlinear associations between each outcome and GA using varying coefficient models, we allowed the location, scale, and skewness parameters to vary linearly with GA within the cohorts (GA:cohort interaction). There was no evidence for differences in the kurtosis parameter between the two cohorts at any GA. Therefore, we modelled the tau parameter across both cohorts. We checked the model assumptions by different diagnostic plots (residuals *vs.* fitted, residuals *vs.* covariates, QQ plots, worm plots). The plots revealed a very good fit, justifying the linearity assumption. No variance inflation was present when modelling the outcomes dependent on GA for both cohorts. To calculate standard deviation scores using our results, the following formula(s) can be used:



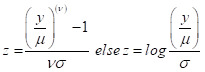



These are good approximations for the true, four-parameter distribution [26]. One can also use the respective *R* functions from the gamlss package (pBCPE(), qBCPE()), or the sds() function from the childsds package. We have completed the STROBE checklist for this work.

## RESULTS

### Description of the Rwanda and the Leipzig cohorts

In Rwanda, we aimed to include women in the second trimester and gestational weeks ranged from 13 to 28, whereas in Leipzig, the aim was to enrol in gestational week 24, and actual enrolment ranged from week 22 to 29 (**Table 1**; Table S1 in the **Online Supplementary Document**). Similarly, for the third trimester assessment, the GAs in Rwanda spans a more extended period (25–41 weeks) than in Leipzig, where the aim for follow-up was week 36 and ranged from 32 to 39 weeks in the data.

**Table 1:** Description of both samples

	Rwanda		Leipzig		*P*-value	
	**n**	**MD (10–90 percentile)**	**n**	MD* (10–90 percentile)	**Location†**	**Spread**
**Maternal age (second trimester)**	303	28.0 (21.0–38.0)	623	30.0 (25.0–37.0)	<0.0001	<0.0001
**GA**						
Second trimester	303	20.0 (15.2–24.2)	623	24.1 (23.1–25.9)	<0.0001	<0.0001
Third trimester	303	32.8 (28.0–36.9)	623	35.4 (35.0–36.1)	<0.0001	<0.0001
**MUAC**						
Second trimester	303	25.4 (22.9–29.4)	614	28.2 (24.8–33.3)	<0.0001	<0.0001
Third trimester	302	25.7 (23.1–29.7)	176	29.1 (25.2–34.0)	<0.0001	0.0001
**Gravidity, %**						
0	70	23.1	293	47.0	<0.0001	
1	80	26.4	201	32.3		
2	62	20.5	74	11.9		
3	36	11.9	32	5.2		
≥4	55	18.2	23	3.7		

MD – median, MUAC – mid-upper arm circumference, GA – gestational age

*Proportion for gravidity.

†*P*-value of the Cochran-Armitage test for trend for gravidity.

### Description of metabolic profiles over gestation and across the cohort

**Figure 1:**
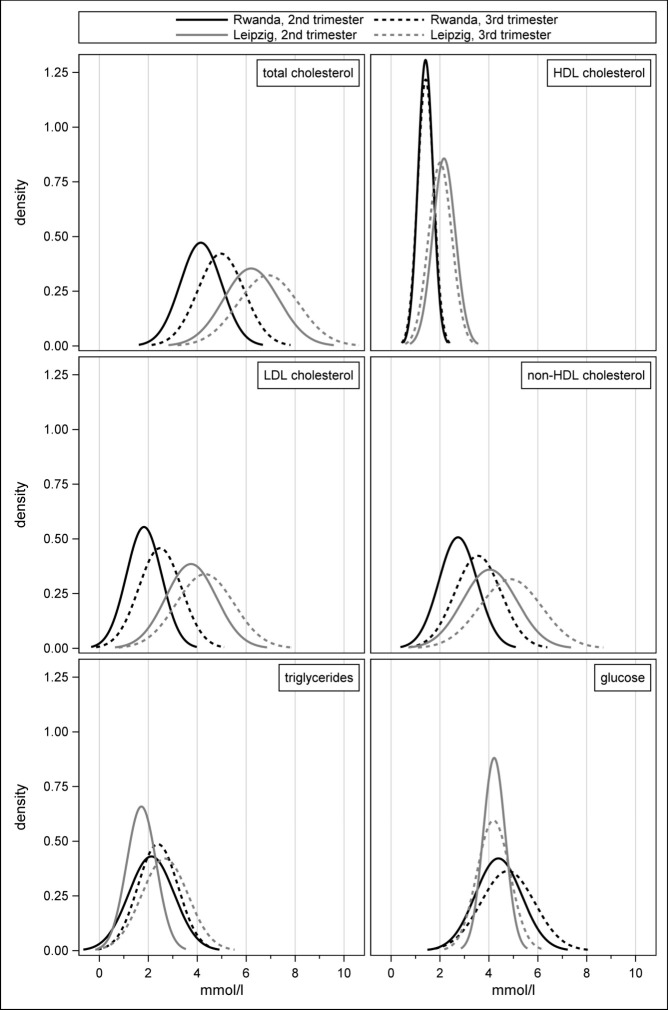
Distribution of the metabolic biomarkers during the second and third trimesters in Rwanda and Leipzig.

For TC, LDL, and non-HDL, there were clear increases from the second to the third trimester, both in Rwanda and Leipzig, while the distributions were shifted to lower concentrations in Rwanda (**Figure 1**). The latter was also true for HDL, which remained consistently low from the second to the third trimester in Rwanda (*β =* 0.01; *P* = 0.56), while Leipzig exhibited a significant decrease (*β* = −0.16; *P* < 0.0001; interaction *P* < 0.0001). Consequently, TC/HDL ratios were found to be comparable between the two cohorts (Table S2 in the **Online Supplementary Document**).

For TG, there was a clear increase from the second to the third trimester in Leipzig (*β =* 0.97; *P* < 0.0001) while this was less pronounced in Rwanda (*β =* 0.27; *P* < 0.0001) (**Table 2**). When comparing the two cohorts, the second-trimester concentrations were higher in Rwanda than in Leipzig (*β =* –0.61; *P* < 0.0001) (**Table 3**). This observation remained even when the analysis was restricted to those who reported fasting within the last eight hours (*β =* –0.47; *P* < 0.001). However, for the third trimester, there was no significant difference in TG concentration between both cohorts (*β =* 0.11; *P* = 0.36; interaction *P* < 0.0001) (Table S12 in the **Online Supplementary Document**).

**Table 2:** Comparison of the metabolic biomarkers across trimesters for each cohort

	Crude			Adjusted*		
	**b†**	**SE**	***P*-value**	**b†**	**SE**	***P*-value**
**Rwanda**						
TC	0.83	0.04	<0.0001	0.83	0.04	<0.0001
HDL	0.01	0.01	0.5630	0.01	0.01	0.5667
LDL	0.65	0.04	<0.0001	0.65	0.04	<0.0001
non-HDL	0.82	0.03	<0.0001	0.82	0.03	<0.0001
TG	0.27	0.06	<0.0001	0.27	0.06	<0.0001
Glucose	0.38	0.08	<0.0001	0.38	0.08	<0.0001
Fasting state						
*TG*	0.33	0.13	0.0165	0.33	0.13	0.0174
*Glucose*	0.48	0.16	0.0042	0.46	0.16	0.0056
**Leipzig**						
TC	0.70	0.03	<0.0001	0.70	0.03	<0.0001
HDL	−0.16	0.01	<0.0001	−0.16	0.01	<0.0001
LDL	0.57	0.03	<0.0001	0.57	0.03	<0.0001
non-HDL	0.85	0.03	<0.0001	0.86	0.03	<0.0001
TG	0.97	0.03	<0.0001	0.97	0.03	<0.0001
Glucose	−0.03	0.03	0.2407	−0.03	0.03	0.2638
Fasting state						
*TG*	0.91	0.03	<0.0001	0.91	0.03	<0.0001
*Glucose*	−0.12	0.02	<0.0001	−0.12	0.02	<0.0001

HDL – high-density lipoprotein cholesterol, LDL – low-density lipoprotein, SE – standard error, TC – total cholesterol, TG – triglycerides

*Adjusted for gestational age (second trimester), time between repeated measurements, maternal age, mid-upper arm circumference (second trimester), and gravidity.

†b: mean difference comparing third to second trimester.

**Table 3:** Comparison of the metabolic biomarkers across both cohorts for each trimester

		Crude			Adjusted*		
		**b†**	**SE**	***P*-value**	**b†**	**SE**	***P*-value**
**Second trimester**							
TC		2.06	0.07	<0.0001	1.60	0.12	<0.0001
HDL		0.76	0.03	<0.0001	0.73	0.05	<0.0001
LDL		1.92	0.07	<0.0001	1.64	0.11	<0.0001
non-HDL		1.30	0.07	<0.0001	0.88	0.12	<0.0001
TG		−0.42	0.05	<0.0001	−0.61	0.09	<0.0001
Glucose		−0.17	0.05	0.0002	−0.24	0.07	0.0012
Fasting state							
*TG*		−0.31	0.09	0.0005	−0.47	0.13	0.0004
*Glucose*		0.09	0.06	0.1557	0.14	0.09	0.1105
**Third trimester**							
TC		1.93	0.08	<0.0001	1.86	0.14	<0.0001
HDL		0.59	0.03	<0.0001	0.59	0.05	<0.0001
LDL		1.84	0.08	<0.0001	1.92	0.14	<0.0001
non-HDL		1.34	0.08	<0.0001	1.26	0.15	<0.0001
TG		0.28	0.06	<0.0001	0.11	0.12	0.3616
Glucose		−0.58	0.06	<0.0001	−0.43	0.13	0.0008
Fasting state							
*TG*		0.27	0.12	0.0207	0.20	0.21	0.3424
*Glucose*		−0.51	0.08	<0.0001	−0.53	0.16	0.0010


HDL – high-density lipoprotein cholesterol, LDL – low-density lipoprotein, SE – standard error, TC – total cholesterol, TG – triglycerides

*Adjusted for gestational age (second trimester), time between repeated measurements, maternal age, mid-upper arm circumference (second trimester), and gravidity.

†b: mean difference comparing third to second trimester.

For glucose concentrations, there was no difference across trimesters in Leipzig (β = –0.03; *P* < 0.26), whereas a significant increase was observed in Rwanda (β = 0.38; *P* < 0.0001). Additionally, the Rwandan distributions had a larger spread compared to those in Leipzig. Glucose concentrations were significantly higher in Rwanda than Leipzig for both the second (*β =* –0.24; *P* < 0.001) and third (*β =* –0.43; *P* < 0.001) trimesters. Following restriction to women who were fasting, glucose concentrations decreased significantly from second to third trimester for Leipzig (*β =* –0.12) and the significant increase in Rwanda became more pronounced (*β =* 0.46); the difference between the two cohorts in the second trimester changed direction and lost statistical significance whereas the difference in the third trimester remained.

These differences for all markers remained largely unchanged when restricting both cohorts to women with GAs from week 22 to 27 (second trimester) and 33 to 38 (third trimester) (Tables S3–S5 in the **Online Supplementary Document**).

### National reference ranges for the metabolites across the trimester

**Figure 2:**
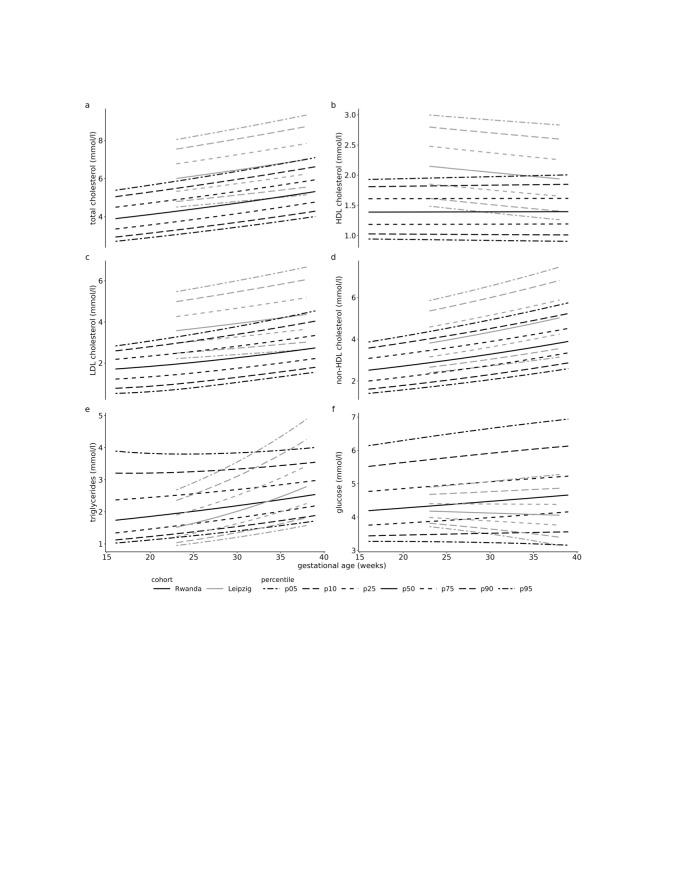
Percentile charts for the metabolic biomarkers.

The Rwandan cohort showed lower ranges for TC during the second trimester, with 95th percentile values of 5.93 mmol/L or higher, and for HDL cholesterol, with second-trimester 5th percentile values up to 0.94 mmol/L or higher (**Figure 2**; Tables S6 and S7 in the **Online Supplementary Document**). In contrast, TG levels were slightly higher in the Rwandan cohort across both trimesters (Table S10 in the **Online Supplementary Document**). For glucose levels, the 75th percentile in the Rwandan cohort exceeded 5.1 mmol/L during the third trimester, while this threshold was only exceeded at the 95th percentile in the Leipzig cohort (Table S11 in the **Online Supplementary Document**).

## DISCUSSION

This study comparing maternal metabolic profiles of pregnant women shows that lipid, lipoprotein, and glucose concentrations vary by geographic location, suggesting differences in ancestry, ethnicity, income, and living conditions, as well as by stage of pregnancy.

During a healthy pregnancy, concentrations of lipids and lipoproteins increase across trimesters [27,28]. In the current study, we found clear increases in TC, LDL, and non-HDL concentrations from the second to the third trimester, except for HDL, which remained equally low in the Rwandan cohort and decreased significantly in the Leipzig cohort. For both trimesters, concentrations in these metabolites were significantly higher in Leipzig than in Rwanda, even after controlling for gestational and maternal age, time between repeated measurements, second-trimester MUAC, and gravidity. These differences confirm findings from previous studies [13,15], indicating a more favourable lipid and lipoprotein profile in Africa than European pregnant women.

### Dyslipidemia and cardiometabolic risk during pregnancy

However, our TG findings do not support this favourable pattern in African populations, as we found significantly higher concentrations for Rwanda compared with Leipzig during the second trimester and no differences during the third trimester. This finding contradicts observations from a previous study conducted between 2008 and 2010, which demonstrated lower TG at both the second (week 15) and third (week 28) trimester of pregnancy for immigrant women of African ethnic origin compared with women of (Eastern and Western) European ethnic origin in Norway [16]. More recent evidence suggests significant variations in TG distributions across populations from different African ancestries and environments [12,29]. For instance, in a recent comprehensive analysis of non-pregnant individuals, East Africans exhibited the highest concentration of TG, followed by Europeans, whereas West African individuals displayed the lowest levels [29].

TG levels also depend on the concentrations of other lipids. For instance, HDL plays a role in clearing TG-rich lipid particles, which is why TG and HDL levels are typically inversely correlated [30]. The favourable pattern of lower TG and higher HDL has been frequently observed in non-pregnant [31,32] and pregnant [13,15] individuals of African descent compared with Caucasians. However, the results of our study do not corroborate these findings. In the Rwandan cohort, TG was higher in the second trimester and similar to that in Leipzig in the third trimester, coinciding with lower HDL in both trimesters. Furthermore, other studies have not consistently observed this pattern of low TG and high HDL [29,33,34]. While TG concentrations vary among individuals of different African and European ancestry in these studies, HDL did not exhibit such variations.

Moreover, dyslipidemia and cardiometabolic risk factors are emerging as a significant public health problem in Africa [35]. During pregnancy, irregular lipid and lipoprotein levels are inherent, yet they increase the likelihood of various adverse maternal and fetal outcomes [36–38], including an elevated risk for cardiovascular disease (CVD) for mother and child even in later life [39–41]. According to a recent study among pregnant women in Nigeria, the prevalence of dyslipidemia was 69.6% during the second and 91.8% during the third trimester [42]. More than 80% of these women were at high risk of developing CVD. The higher TG and lower HDL concentrations observed in the Rwandan cohort may support the emerging burden of chronic conditions and risk factors in low-income settings, including those in Africa. However, the lipid measures used for predicting CVD risk differ in the literature [43–46], which raises the potential of contradictory risk assessments. For instance, while significantly lower levels of non-HDL in Rwanda suggest an increased risk for CVD, the TC/HDL ratio, comparable between Rwanda and Leipzig, indicates no elevated risk. While non-HDL is the preferred marker for CVD risk in European populations, the TC/HDL ratio may be a more appropriate marker for other populations.

Although not all the findings can be directly applied or transferred to our samples of pregnant women, it remains plausible to assume that the observed TG and HDL distributions might adhere to an ancestry-specific pattern, resulting in higher TG and lower HDL levels for Rwanda compared with Leipzig. These observations again underscore the necessity for national reference ranges, primarily to ensure patient safety and avoid bias in future research endeavours.

### Emerging burden of GDM

During a healthy pregnancy, fasting blood plasma glucose levels typically decline across trimesters [47] due to increased fetoplacental glucose use [48,49]. In the current study, there were no significant alterations in fasting glucose concentration across the trimesters for Leipzig, likely due to proper screening for and management of excessive glucose levels. In contrast, the Rwandan cohort significantly increased from the second to the third trimester. According to recommendations from the International Association of Diabetes and Pregnancy Study Group, GDM is defined by the presence of fasting plasma glucose values ≥5.1 mmol/L between 24 and 28 weeks of gestation [50]. In the current study, this cut-off was exceeded during the third trimester for 24% of the women in the Rwandan cohort compared to a significantly lower prevalence of 7% for Leipzig. Studies on GDM in Rwanda, employing various methods and diagnostic criteria, have reported prevalence rates ranging between 3.2% [51] and 8.3% [52,53]. Across Africa, a recent meta-analysis found that the overall prevalence is estimated to be 5%, which is notably higher compared to certain high-income countries [54].

Ancestral differences in the risk for GDM have been widely reported [55–57]. For instance, a large cohort study in the Netherlands revealed a five to 7-fold higher risk of GDM in women of sub-Saharan African descent compared to Dutch women [56]. This effect was independent of body mass index (BMI), a similar finding to our study, where glucose concentration remained significantly elevated for Rwanda, even after adjusting for differences in MUAC. One possible explanation could involve proposed ancestral variations in insulin resistance and response [58]. For example, African populations with standard glucose tolerance show higher insulin resistance than Europeans [59,60]. Moreover, as they progress from standard glucose tolerance to impaired fasting blood glucose and type 2 diabetes, insulin production declines more rapidly in Africans than in Europeans [58]. In line with this, a study on pregnant women of different ancestries showed that Africans experience higher insulin resistance during the second and third trimesters, along with lower insulin production during the third trimester compared to Caucasians [61]. Notably, in that study and during both trimesters, no differences in fasting glucose were observed between Africans and Caucasians.

Typically, it is assumed that when there is increased insulin resistance, the body responds with higher insulin secretion to compensate and keep blood glucose levels within a normal range [59]. This implies that potential impairments in insulin resistance in Rwanda during early or mid-pregnancy may have subsequently led to the significantly elevated glucose concentrations observed during the later stage of pregnancy, when compensatory insulin production by beta cells becomes insufficient. In Caucasians with insulin resistance, TG concentration is typically expected to be elevated. However, in individuals of African descent, a characteristic lipid profile for insulin resistance includes low HDL and normal rather than elevated TG concentrations [62,63].

Although these findings cannot be directly transferred to our samples of pregnant women, it remains plausible to assume that, despite similar TG levels during the third trimester, individuals of the Rwandan sample may have been insulin resistant. In conjunction with these potential biological predispositions, considering that 54% of individuals from sub-Saharan Africa live with undiagnosed diabetes and that Rwanda faces a shortage of trained health professionals [64], along with limited knowledge and biased perceptions of diabetes [65], it is possible that women in the Rwandan sample might have remained undiagnosed with diabetes.

### National reference ranges

The metabolic differences in pregnant women, as demonstrated above and supported by results from previous studies, indicate that lipids and lipoproteins might be multifactorial and influenced by ethnic and geographical factors. Thus, there is a need for national reference ranges. In this way, the calculated reference ranges of the current study, compared with those already established, show mixed results.

Compared to studies from China [22,23] and Poland [21], the Leipzig cohort showed similar or slightly higher reference limits for TC, HDL, and LDL in both the second and third trimesters. In contrast, the Rwandan cohort exhibited lower ranges for TC (*e.g.* second trimester: up to 1.4 mmol/L) and HDL (*e.g.* second trimester: up to 0.8 mmol/L) compared to the study by Wang et al. [22]. It also showed significantly lower TC (up to 2.0 mmol/L) and LDL ranges (up to 2.1 mmol/L) compared to a Polish study [21]. Further, for HDL, Rwanda exhibited similarly low values in the 5th percentile but slightly higher values (up to 0.6 mmol/L) in the 95th percentile.

For TG, Leipzig had slightly lower to similar values compared to the study by Wang et al. [22], while Rwanda showed slightly higher values across both trimesters. However, both Rwanda (up to 2.3 mmol/L) and Leipzig (up to 1.3 mmol/L) had significantly lower concentrations during both trimesters compared to the study by Wu et al. [23]. Compared to the Polish sample, both Rwanda and Leipzig showed reference ranges similar to or slightly higher than those during the second trimester [21]. During the third trimester, values were similar to lower reference ranges, and for Leipzig, they were highly dependent on GA.

Regarding glucose levels, 5.1 mmol/L was exceeded during the third trimester at the 75th percentile for the Rwandan cohort and at the 95th percentile for the Leipzig cohort. A study from Nigeria reported glucose ranges of 3.4–6.4 mmol/L for the second trimester and 4.0–6.1 mmol/L for the third trimester [66]. Rwandan values were similar to those from Nigeria in the second trimester but higher in the third trimester, reaching up to 0.7 mmol/L above the Nigerian values at the 95th percentile. In contrast, Leipzig showed significantly lower glucose concentrations than Nigeria in both trimesters, especially at the 95th percentile. A study from China reported a reference range of 3.3–7.4 mmol/L during the third trimester, which is similar to the values observed in Rwanda and Nigeria [67]. Notably, the Chinese study [67] analysed non-fasting blood samples, while the Nigerian study [66] included women attending antenatal care, with fasting status not specified. Thus, as values may vary based on factors such as fasting status and regional demographics, establishing reference ranges in other African regions is essential [68]. As there is no consensus about cut-offs for pregnant women, conclusions about potential maternal and fetal risks indicated by these reference ranges are difficult. Since metabolic risk profiles in non-pregnant individuals vary by ancestry [62,63], further studies should evaluate risk profiles during pregnancy based on region-specific reference ranges.

### Limitations

Residual confounding by other factors, such as biochemical measurement methods, dietary habits, or data collection methods, may have contributed to substantial variation and limited current findings. Moreover, due to the rare use of ultrasound during gestation in Rwanda, compared to Germany, where it is standard, GAs may be less precise in the Rwandan cohort than in the Leipzig cohort. A further limitation is that it was not possible to include random terms in the GAMLSS models, as the resulting group sizes and number of groups led to non-convergence and non-identifiability.

It is widely recognised that glucose levels in plasma are generally 10–15% higher than those measured in whole blood [69], which, in contrast to our findings, would suggest lower glucose concentrations for Rwanda, where we used capillary whole blood. Also, point-of-care testing systems have lower accuracy than laboratory-based methods, which may explain the wider data distribution. Nevertheless, a separate study has demonstrated strong associations between point-of-care testing and fasting plasma glucose and a satisfactory diagnostic performance for GDM [70]. Thus, it is more plausible that other factors, such as potential ancestral differences in glucose metabolism and/or undiagnosed diabetes in the Rwandan sample, have contributed to the variation in the observed glucose concentration.

Furthermore, circulating glucose [71,72], TG levels are highly influenced by food intake. For this reason, participants were instructed to fast for eight hours before each examination. However, this seemed particularly challenging for the Rwandan cohort, with many individuals, both in the second (35%) and the third (70%) trimester, not adhering to fasting requirements. This might reflect logistical challenges, including the long distances pregnant individuals had to walk to reach the medical assessment centres. Nevertheless, even after excluding these non-fasting individuals, the overall findings of this study remained unchanged, although there was an increase in the spread of glucose and TG distributions, underscoring potential associations to food intake.

Another limitation of this study, therefore, is the lack of adjustment for dietary habits. While in Western populations, a diet rich in high-fat dairy products, meat, and processed food is prevalent [73], the primary sources of daily food in Rwanda are vegetable products [74]. Lower HDL concentration, as observed in the Rwandan cohort, has previously been attributed to low dietary protein intake in samples of South African individuals [33]. A Western-style diet is associated with abnormal lipid concentrations, which we did not observe in the Leipzig cohort compared with the Rwandan cohort. Although we cannot rule out the possibility that different dietary patterns may have influenced current results, it appears likely that other factors also contributed to these outcomes.

For instance, divergent levels of TG among ancestral groups have been partially linked to different fat deposition patterns (visceral *vs.* subcutaneous). Women of African descent tend to accumulate more subcutaneous fat [75]. As a result, the correlation between TG levels and BMI appears to be more pronounced in European populations than in African descent. In addition, BMI is highly correlated with GA. Given the differences in GA between the two samples, using BMI as a control variable might have introduced additional confounding. To address this, we used MUAC, which shows only minimal change during pregnancy [76,77] and thus indicates pre-pregnancy body fat. Although we lack data on MUAC (and BMI) before pregnancy in the Rwandan sample, using MUAC most likely corrected for potential confounding due to different body fat levels to a large extent. Nevertheless, to mitigate any potential bias related to fat deposition patterns in multi-ancestral studies, future research could consider using intra-individual differences in total fat mass.

### Strengths

Despite its limitations, the current study provides multiple insights into differences in metabolic biomarkers and emerging chronic conditions and risk factors in African settings. This study appears to be the first representative investigation of lipid, lipoprotein, and glucose concentrations in pregnant women from Rwanda, offering a comparison to a Caucasian population. Until now, most studies on pregnant women in Rwanda have focused on other parameters, such as glucose, particularly the prevalence of GDM [51-53], which is an increasing health concern in Africa. Our study expands this literature by providing contemporary data on glucose changes and other metabolic biomarkers.

A key strength of the current study is the establishment of the first maternal reference ranges for these metabolic biomarkers in Rwanda. In general, especially in sub-Saharan Africa, published reference ranges are sparse regardless of the topic [68]. Our data, when compared with other studies on African individuals, suggest that reference ranges should be region-specific to support patient care and guide further clinical research studies appropriately. Consequently, the results of this study provide practitioners with reliable measures for pregnancy monitoring in Rwanda, which can significantly contribute to maternal and fetal health care. In this regard, the current study highlights the importance of longitudinal data in understanding the dynamic nature of metabolic changes during pregnancy. Such data are crucial for providing precise insights for clinical practice, particularly concerning the potential needs or risks of pregnant women in the respective trimesters.

## CONCLUSIONS

The emerging burden of altered lipid and glucose metabolism-associated diseases in the Rwandan cohort and Leipzig underscores the urgent need for attention and proactive measures to mitigate their impact on maternal and child health. Addressing the emerging burden of GDM in Africa is crucial for improving maternal and child health outcomes across the continent. This should include implementing targeted interventions, regular screening during pregnancy, and raising awareness about the condition. Moreover, national reference ranges are also essential in other African regions to facilitate pregnancy monitoring, assess potential risks, and guide the design of prospective studies.

## Additional material


Online Supplementary Document

## References

[1] Zeng Z Liu F Li S Metabolic Adaptations in Pregnancy: A Review. Ann Nutr Metab. 2017;70:59–65. 10.1159/000459633 28297696

[2] Di Cianni G Miccoli R Volpe L Lencioni C Del Prato S Intermediate metabolism in normal pregnancy and in gestational diabetes. Diabetes Metab Res Rev. 2003;19:259–70. 10.1002/dmrr.390 12879403

[3] Trivett C Lees ZJ Freeman DJ Adipose tissue function in healthy pregnancy, gestational diabetes mellitus and pre-eclampsia. Eur J Clin Nutr. 2021;75:1745–56. 10.1038/s41430-021-00948-9 34131300 PMC8636251

[4] Edison RJ Berg K Remaley A Kelley R Rotimi C Stevenson RE Adverse Birth Outcome Among Mothers With Low Serum Cholesterol. Obstet Gynecol Surv. 2008;63:81–2. 10.1097/01.ogx.0000300468.11718.4a 17908758

[5] Wang C Zhu W Wei Y Su R Feng H Hadar E The associations between early pregnancy lipid profiles and pregnancy outcomes. J Perinatol. 2017;37:127–33. 10.1038/jp.2016.191 27787507

[6] Kitajima M Oka S Yasuhi I Fukuda M Rii Y Ishimaru T Maternal serum triglyceride at 24–32 weeks’ gestation and newborn weight in nondiabetic women with positive diabetic screens. Obstet Gynecol. 2001;97:776–80. 10.1016/S0029-7844(01)01328-X 11339933

[7] Yue CY Ying C Epidemiological analysis of maternal lipid levels during the second trimester in pregnancy and the risk of adverse pregnancy outcome adjusted by pregnancy BMI. J Clin Lab Anal. 2018;32:e22568. 10.1002/jcla.22568 29774596 PMC6817034

[8] Enquobahrie DA Williams MA Butler CL Frederick IO Miller RS Luthy DA Maternal plasma lipid concentrations in early pregnancy and risk of preeclampsia. Am J Hypertens. 2004;17:574–81. 10.1016/j.amjhyper.2004.03.666 15233976

[9] Riskin-Mashiah S Younes G Damti A Auslender R First-trimester fasting hyperglycemia and adverse pregnancy outcomes. Diabetes Care. 2009;32:1639–43. 10.2337/dc09-0688 19549728 PMC2732138

[10] HAPO Study Cooperative Research Group Metzger BE Lowe LP Dyer AR Trimble ER Chaovarindr U Hyperglycemia and adverse pregnancy outcomes. N Engl J Med. 2008;358:1991–2002. 10.1056/NEJMoa0707943 18463375

[11] Zhao D Liu D Shi W Shan L Yue W Qu P Association between Maternal Blood Glucose Levels during Pregnancy and Birth Outcomes: A Birth Cohort Study. Int J Environ Res Public Health. 2023;20:2102. 10.3390/ijerph20032102 36767469 PMC9915873

[12] Bentley AR Rotimi CN Interethnic Differences in Serum Lipids and Implications for Cardiometabolic Disease Risk in African Ancestry Populations. Glob Heart. 2017;12:141–50. 10.1016/j.gheart.2017.01.011 28528248 PMC5582986

[13] Koukkou E Watts GF Mazurkiewicz J Lowy C Ethnic differences in lipid and lipoprotein metabolism in pregnant women of African and Caucasian origin. J Clin Pathol. 1994;47:1105–7. 10.1136/jcp.47.12.1105 7876384 PMC502203

[14] Chen X Scholl TO Stein TP Steer RA Williams KP Maternal Circulating Lipid Profile during Early Pregnancy: Racial/Ethnic Differences and Association with Spontaneous Preterm Delivery. Nutrients. 2017;9:19. 10.3390/nu9010019 28045435 PMC5295063

[15] Schreuder YJ Hutten BA van Eijsden M Jansen EH Vissers MN Twickler MT Ethnic differences in maternal total cholesterol and triglyceride levels during pregnancy: the contribution of demographics, behavioural factors and clinical characteristics. Eur J Clin Nutr. 2011;65:580–9. 10.1038/ejcn.2010.282 21245878

[16] Waage CW Mdala I Stigum H Jenum AK Birkeland KI Shakeel N Lipid and lipoprotein concentrations during pregnancy and associations with ethnicity. BMC Pregnancy Childbirth. 2022;22:246. 10.1186/s12884-022-04524-2 35331154 PMC8953044

[17] Scholl TO Chen X Gaughan C Smith WK Influence of maternal glucose level on ethnic differences in birth weight and pregnancy outcome. Am J Epidemiol. 2002;156:498–506. 10.1093/aje/kwf080 12225997

[18] Phillipou G Phillips PJ Racial differences in oral glucose screening test results: establishing race-specific criteria for abnormality in pregnancy. Obstet Gynecol. 1993;82:479–80. 8355957

[19] Lu Y Jia Z Su S Han L Meng L Tang G Establishment of trimester-specific reference intervals of serum lipids and the associations with pregnancy complications and adverse perinatal outcomes: a population-based prospective study. Ann Med. 2021;53:1632–41. 10.1080/07853890.2021.1974082 34498500 PMC8439224

[20] Dathan-Stumpf A Vogel M Jank A Thiery J Kiess W Stepan H Reference intervals of serum lipids in the second and third trimesters of pregnancy in a Caucasian cohort: the LIFE Child study. Arch Gynecol Obstet. 2019;300:1531–9. 10.1007/s00404-019-05342-2 31667609

[21] Piechota W Staszewski A Reference ranges of lipids and apolipoproteins in pregnancy. Eur J Obstet Gynecol Reprod Biol. 1992;45:27–35. 10.1016/0028-2243(92)90190-A 1618359

[22] Wang C Kong L Yang Y Wei Y Zhu W Su R Recommended reference values for serum lipids during early and middle pregnancy: a retrospective study from China. Lipids Health Dis. 2018;17:246. 10.1186/s12944-018-0885-3 30382875 PMC6211477

[23] Wu L Wu Q Li Q Cao S Zhang Y Liu Y Consecutive reference intervals for biochemical indices related to serum lipid levels and renal function during normal pregnancy. BMC Pregnancy Childbirth. 2022;22:642. 10.1186/s12884-022-04960-0 35971117 PMC9377122

[24] Poulain T Baber R Vogel M Pietzner D Kirsten T Jurkutat A The LIFE Child study: a population-based perinatal and pediatric cohort in Germany. Eur J Epidemiol. 2017;32:145–58. 10.1007/s10654-016-0216-9 28144813

[25] Rigby RA Stasinopoulos DM Smooth centile curves for skew and kurtotic data modelled using the Box–Cox power exponential distribution. Stat Med. 2004;23:3053–76. 10.1002/sim.1861 15351960

[26] Rdocumentation. BCPE: Box-Cox Power Exponential distribution for fitting a GAMLSS. 2017. Available: https://www.rdocumentation.org/packages/gamlss.dist/versions/6.1-1/topics/BCPE. Accessed: 25 June 2025.

[27] Wild R, Feingold KR. Effect of Pregnancy on Lipid Metabolism and Lipoprotein Levels. In: Feingold KR, Anawalt B, Blackman MR, Blackman MR, Boyce A, Chrousos G, et al., editors. Endotext. South Dartmouth, Massachusetts: MDText.com, Inc.; 2000. p. 2000-2022.

[28] Bao W Dar S Zhu Y Wu J Rawal S Li S Plasma concentrations of lipids during pregnancy and the risk of gestational diabetes mellitus: A longitudinal study. J Diabetes. 2018;10:487–95. 10.1111/1753-0407.12563 28436169 PMC5837900

[29] Meeks KAC Bentley AR Agyemang C Galenkamp H van den Born BH Hanssen NMJ Ancestral and environmental patterns in the association between triglycerides and other cardiometabolic risk factors. EBioMedicine. 2023;91:104548. 10.1016/j.ebiom.2023.104548 37004336 PMC10102222

[30] Kolovou GD Anagnostopoulou K Pilatis ND Salpea KD Hoursalas IS Petropoulos I Fasting serum triglyceride and high-density lipoprotein cholesterol levels in patients intended to be treated for dyslipidemia. Vasc Health Risk Manag. 2005;1:155–61. 10.2147/vhrm.1.2.155.64079 17315402 PMC1993943

[31] Ervin RB Prevalence of metabolic syndrome among adults 20 years of age and over, by sex, age, race and ethnicity, and body mass index: United States, 2003-2006. Natl Health Stat Report. 2009;13:1–7. 19634296

[32] Sumner AE Cowie CC Ethnic differences in the ability of triglyceride levels to identify insulin resistance. Atherosclerosis. 2008;196:696–703. 10.1016/j.atherosclerosis.2006.12.018 17254586

[33] Goedecke JH Utzschneider K Faulenbach MV Rizzo M Berneis K Spinas GA Ethnic differences in serum lipoproteins and their determinants in South African women. Metabolism. 2010;59:1341–50. 10.1016/j.metabol.2009.12.018 20096899

[34] Jennings CL Lambert EV Collins M Joffe Y Levitt NS Goedecke JH Determinants of insulin-resistant phenotypes in normal-weight and obese Black African women. Obesity (Silver Spring). 2008;16:1602–9. 10.1038/oby.2008.233 18421268

[35] Obsa MS Ataro G Awoke N Jemal B Tilahun T Ayalew N Determinants of Dyslipidemia in Africa: A Systematic Review and Meta-Analysis. Front Cardiovasc Med. 2022;8:778891. 10.3389/fcvm.2021.778891 35284497 PMC8904727

[36] Tesfa E Nibret E Munshea A Maternal lipid profile and risk of pre-eclampsia in African pregnant women: A systematic review and meta-analysis. PLoS One. 2020;15:e0243538. 10.1371/journal.pone.0243538 33362205 PMC7757810

[37] Wang J Moore D Subramanian A Cheng KK Toulis KA Qiu X Gestational dyslipidaemia and adverse birthweight outcomes: a systematic review and meta-analysis. Obes Rev. 2018;19:1256–68. 10.1111/obr.12693 29786159

[38] Mauri M Calmarza P Ibarretxe D Dyslipemias and pregnancy, an update. Clin Investig Arterioscler. 2021;33:41–52. 33309071 10.1016/j.arteri.2020.10.002

[39] Palinski W Effect of Maternal Cardiovascular Conditions and Risk Factors on Offspring Cardiovascular Disease. Circulation. 2014;129:2066–77. 10.1161/CIRCULATIONAHA.113.001805 24842934 PMC4053195

[40] Higa R Jawerbaum A Intrauterine effects of impaired lipid homeostasis in pregnancy diseases. Curr Med Chem. 2013;20:2338–50. 10.2174/0929867311320180005 23521676

[41] Kramer CK Campbell S Retnakaran R Gestational diabetes and the risk of cardiovascular disease in women: a systematic review and meta-analysis. Diabetologia. 2019;62:905–14. 10.1007/s00125-019-4840-2 30843102

[42] Saliu MA Salihu A Mada SB Owolabi OA Dyslipidaemia-related cardiovascular risk among pregnant women attending Aminu Kano Teaching Hospital Kano: A longitudinal study. J Taibah Univ Med Sci. 2021;16:870–7. 10.1016/j.jtumed.2021.07.004 34899132 PMC8626804

[43] Damen JAAG Hooft L Schuit E Debray TP Collins GS Tzoulaki I Prediction models for cardiovascular disease risk in the general population: systematic review. BMJ. 2016;353:i2416. 10.1136/bmj.i2416 27184143 PMC4868251

[44] Visseren FLJ Mach F Smulders YM Carballo D Koskinas KC Bäck M 2021 ESC Guidelines on cardiovascular disease prevention in clinical practice. Eur Heart J. 2021;42:3227–337. 10.1093/eurheartj/ehab484 34458905

[45] Calling S Johansson SE Wolff M Sundquist J Sundquist K Total cholesterol/HDL-C ratio versus non-HDL-C as predictors for ischemic heart disease: a 17-year follow-up study of women in southern Sweden. BMC Cardiovasc Disord. 2021;21:163. 10.1186/s12872-021-01971-1 33820540 PMC8020530

[46] Lemieux I Lamarche B Couillard C Pascot A Cantin B Bergeron J Total cholesterol/HDL cholesterol ratio vs LDL cholesterol/HDL cholesterol ratio as indices of ischemic heart disease risk in men: the Quebec Cardiovascular Study. Arch Intern Med. 2001;161:2685–92. 10.1001/archinte.161.22.2685 11732933

[47] Riskin-Mashiah S Damti A Younes G Auslander R Normal fasting plasma glucose levels during pregnancy: a hospital-based study. J Perinat Med. 2011;39:209–11. 10.1515/jpm.2010.142 21241203

[48] Hay WW Jr Placental-fetal glucose exchange and fetal glucose metabolism. Trans Am Clin Climatol Assoc. 2006;117:321–39. 18528484 PMC1500912

[49] Desoye G Nolan CJ The fetal glucose steal: an underappreciated phenomenon in diabetic pregnancy. Diabetologia. 2016;59:1089–94. 10.1007/s00125-016-3931-6 26995651 PMC4861753

[50] International Association of Diabetes and Pregnancy Study Groups Consensus Panel Metzger BE Gabbe SG Persson B Buchanan TA Catalano PA International association of diabetes and pregnancy study groups recommendations on the diagnosis and classification of hyperglycemia in pregnancy. Diabetes Care. 2010;33:676–82. 10.2337/dc10-0719 20190296 PMC2827530

[51] Meharry PM Tengera O Rulisa S Byambu AK Nietert PJ Byiringiro S Prevalence of gestational diabetes mellitus among women attending antenatal care at public health centers in Rwanda. Diabetes Res Clin Pract. 2019;151:252–9. 10.1016/j.diabres.2019.03.035 30946850 PMC6941349

[52] Mapira HT Tumusiime DK Yarasheski K Rujeni N Cade TW Mutimura E Strategy to improve the burden of gestational diabetes in African women: Rwandan perspective. Rwanda J. 2017;4:36–8. 10.4314/rj.v4i1.5F

[53] Niyibizi JB Safari F Ahishakiye JB Habimana JB Mapira H Mutuku NC Gestational Diabetes Mellitus and Its Associated Risk Factors in Pregnant Women at Selected Health Facilities in Kigali City, Rwanda. J Diabetes Mellitus. 2016;6:269–76. 10.4236/jdm.2016.64028

[54] Macaulay S Dunger DB Norris SA Gestational diabetes mellitus in Africa: a systematic review. PLoS One. 2014;9:e97871. 10.1371/journal.pone.0097871 24892280 PMC4043667

[55] Hedderson M Ehrlich S Sridhar S Darbinian J Moore S Ferrara A Racial/ethnic disparities in the prevalence of gestational diabetes mellitus by BMI. Diabetes Care. 2012;35:1492–8. 10.2337/dc11-2267 22619080 PMC3379591

[56] Rademaker D van Schaijik CI Oostvogels AJJM van Rijn BB Evers I DeVries JH Gestational diabetes mellitus among Sub-Saharan African and Surinamese women in the Netherlands. Diabetes Res Clin Pract. 2020;168:108367. 10.1016/j.diabres.2020.108367 32791160

[57] Jaffe A Giveon S Rubin C Novikov I Ziv A Kalter-Leibovici O Gestational diabetes risk in a multi-ethnic population. Acta Diabetol. 2020;57:263–9. 10.1007/s00592-019-01404-8 31494746 PMC7049543

[58] Kodama K Tojjar D Yamada S Toda K Patel CJ Butte AJ Ethnic Differences in the Relationship Between Insulin Sensitivity and Insulin Response. Diabetes Care. 2013;36:1789–96. 10.2337/dc12-1235 23704681 PMC3661854

[59] Hasson BR Apovian C Istfan N Racial/Ethnic Differences in Insulin Resistance and Beta Cell Function: Relationship to Racial Disparities in Type 2 Diabetes among African Americans versus Caucasians. Curr Obes Rep. 2015;4:241–9. 10.1007/s13679-015-0150-2 26627219

[60] Haffner SM D’Agostino R Saad MF Rewers M Mykkänen L Selby J Increased insulin resistance and insulin secretion in nondiabetic African-Americans and Hispanics compared with non-Hispanic whites. The Insulin Resistance Atherosclerosis Study. Diabetes. 1996;45:742–8. 10.2337/diab.45.6.742 8635647

[61] Chen X Scholl TO Ethnic differences in C-peptide/insulin/glucose dynamics in young pregnant women. J Clin Endocrinol Metab. 2002;87:4642–6. 10.1210/jc.2001-011949 12364450

[62] Sumner AE Vega GL Genovese DJ Finley KB Bergman RN Boston RC Normal triglyceride levels despite insulin resistance in African Americans: role of lipoprotein lipase. Metabolism. 2005;54:902–9. 10.1016/j.metabol.2005.03.001 15988699

[63] Sumner AE “Half the Dyslipidemia of Insulin Resistance” Is the Dsylipidemia of Insulin-Resistant Blacks. Ethn Dis. 2009;19:462–5. 20073149 PMC3482474

[64] National Academies of Sciences, Engineering, and Medicine, Health and Medicine Division; Board on Global Health, Committee on the Evaluation of Strengthening Human Resources for Health Capacity in the Republic of Rwanda Under the President’s Emergency Plan for AIDS Relief (PEPFAR). Evaluation of PEPFAR’s Contribution (2012-2017) to Rwanda’s Human Resources for Health Program. Washington, D.C., USA: National Academies of Sciences; 2020. Available: https://nap.nationalacademies.org/catalog/25687/evaluation-of-pepfars-contribution-2012-2017-to-rwandas-human-resources-for-health-program. Accessed: 13 June 2024.

[65] Mukeshimana MM Nkosi ZZ Communities’ knowledge and perceptions of type two diabetes mellitus in Rwanda: a questionnaire survey. J Clin Nurs. 2014;23:541–9. 10.1111/jocn.12199 23789978

[66] Miri-Dashe T Osawe S Tokdung M Daniel N Choji RP Mamman I Comprehensive reference ranges for hematology and clinical chemistry laboratory parameters derived from normal Nigerian adults. PLoS One. 2014;9:e93919. 10.1371/journal.pone.0093919 24832127 PMC4022493

[67] Jin Y Lu J Jin H Fei C Xie X Zhang J Reference intervals for biochemical, haemostatic and haematological parameters in healthy Chinese women during early and late pregnancy. Clin Chem Lab Med. 2018;56:973–9. 10.1515/cclm-2017-0804 29303769

[68] Price MA Fast PE Mshai M Lambrick M Machira YW Gieber L Region-specific laboratory reference intervals are important: A systematic review of the data from Africa. PLOS Glob Public Health. 2022;2:e0000783. 10.1371/journal.pgph.0000783 36962599 PMC10021479

[69] Kotwal N Pandit A Variability of capillary blood glucose monitoring measured on home glucose monitoring devices. Indian J Endocrinol Metab. 2012;16:S248–51. 10.4103/2230-8210.104052 23565391 PMC3603039

[70] Coetzee A van de Vyver M Hoffmann M Hall DR Mason D Conradie M A comparison between point-of-care testing and venous glucose determination for the diagnosis of diabetes mellitus 6-12 weeks after gestational diabetes. Diabet Med. 2019;36:591–9. 10.1111/dme.13903 30663133

[71] O’Keefe JH Gheewala NM O’Keefe JO Dietary Strategies for Improving Post-Prandial Glucose, Lipids, Inflammation, and Cardiovascular Health. J Am Coll Cardiol. 2008;51:249–55. 10.1016/j.jacc.2007.10.016 18206731

[72] Russell WR Baka A Björck I Delzenne N Gao D Griffiths HR Impact of Diet Composition on Blood Glucose Regulation. Crit Rev Food Sci Nutr. 2016;56:541–90. 10.1080/10408398.2013.792772 24219323

[73] Ushula TW Mamun A Darssan D Wang WYS Williams GM Whiting SJ Dietary patterns and the risk of abnormal blood lipids among young adults: A prospective cohort study. Nutr Metab Cardiovasc Dis. 2022;32:1165–74. 10.1016/j.numecd.2022.01.030 35260316

[74] African Development Bank Group. Rwanda - Food Balance Sheets 2017-2021. 26 September 2023. Available: https://www.afdb.org/en/documents/rwanda-food-balance-sheets-2017-2021. Accessed: 13 June 2024.

[75] Katzmarzyk PT Bray GA Greenway FL Johnson WD Newton RL Jr Ravussin E Racial differences in abdominal depot-specific adiposity in white and African American adults. Am J Clin Nutr. 2010;91:7–15. 10.3945/ajcn.2009.28136 19828714 PMC6443262

[76] Mahaba HM Ismail NA El Teheiwy MM El-Goewily MM Ramadan MS Development of weight gain charts for healthy Egyptian pregnant women. J Egypt Public Health Assoc. 2001;76:369–91. 17216933

[77] López LB Calvo EB Poy MS del Valle Balmaceda Y Cámera K Changes in skinfolds and mid-upper arm circumference during pregnancy in Argentine women. Matern Child Nutr. 2011;7:253–62. 10.1111/j.1740-8709.2009.00237.x 21689268 PMC6860578

