# Supplementary material for: Differences in metabolic adaptations during mid and late pregnancy: a comparative cohort study between Rwanda and Germany
Source: J Glob Health. 2025 Jul 11;15:04206. doi: 10.7189/jogh.15.04206 (PMC12247662; doi:10.7189/jogh.15.04206)
Supplement: Online Supplementary Document [file jogh-15-04206-s001.pdf]

Supplement to: Amberbir A, Ordnung M, Ishimwe SMC, Biemann R, Vogel M, Kiess W, Körner A, Nigatu B, Bazimya D, Uwizeyimana T, Niyibizi JB, Seifu D, Bekele A, Genuneit J. Differences in metabolic adaptations during mid and late pregnancy: a comparative cohort study between Rwanda and Germany. J Glob Health. 2025;15:04206.

Supplementary Table 1. Description of the study populations of fasting women at both 2nd and 3rd trimester

Variable	Rwanda		Leipzig			
	N	Median[1] (10th; 90th)	N	Median[1] (10th; 90th)	p-value for location[2]	p-value for spread
Maternal age (2nd trimester)	63	28.0 (20.0; 39.0)	512	30.0 (25.0; 37.0)	0.0032	<.0001
Gestational age						
2nd trimester	63	20.2 (15.4; 24.1)	512	24.1 (23.1; 25.7)	<.0001	<.0001
3rd trimester	63	33.4 (28.1; 37.3)	512	35.4 (35.0; 36.1)	<.0001	<.0001
Mid-upper arm circumference						
2nd trimester	63	25.5 (23.0; 30.5)	505	28.1 (25.0; 33.1)	<.0001	0.6678
3rd trimester	63	25.7 (23.1; 29.7)	156	29.2 (25.7; 33.9)	<.0001	0.1500
Gravidity (%)						
0	16	25.4	251	49	<.0001	
1	12	19	159	31.1		
2	12	19	58	11.3		
3	6	9.5	26	5.1		
4 or more	17	27	18	3.5		

Note. Displayed are the median and the 10th and 90th percentiles for the variables.

[1] Proportion for gravidity.

[2] p-value of the Cochran-Armitage test for trend for gravidity.

Supplementary Table 2. Description of the metabolic biomarkers in mmol/l

Biomarker	Trimester	Rwanda			Leipzig		
		N	Mean (STD)	Median (10th; 90th)	N	Mean (STD)	Median (10th; 90th)
TC	2nd	301	4.1 (0.9)	4.1 (3.1; 5.3)	614	6.2 (1.1)	6.1 (4.8; 7.6)
	3rd	302	5.0 (0.9)	4.9 (3.8; 6.3)	622	6.9 (1.2)	6.8 (5.4; 8.4)
HDL	2nd	296	1.4 (0.3)	1.4 (1.0; 1.9)	614	2.2 (0.5)	2.1 (1.6; 2.8)
	3rd	298	1.4 (0.3)	1.4 (1.1; 1.8)	622	2.0 (0.5)	2.0 (1.4; 2.6)
LDL	2nd	284	1.8 (0.7)	1.8 (0.9; 2.8)	614	3.7 (1.0)	3.7 (2.5; 5.1)
	3rd	290	2.5 (0.9)	2.4 (1.4; 3.7)	622	4.3 (1.2)	4.2 (2.9; 5.8)
non-HDL	2nd	296	2.7 (0.8)	2.8 (1.7; 3.9)	614	4.0 (1.1)	3.9 (2.7; 5.4)
	3rd	298	3.6 (0.9)	3.5 (2.4; 4.9)	622	4.9 (1.3)	4.8 (3.3; 6.4)
TC/HDL ratio	2nd	296	3.0 (0.8)	2.9 (2.1; 4.0)	614	3.0 (0.8)	2.9 (2.1; 3.9)
	3rd	298	3.7 (1.0)	3.5 (2.5; 5.3)	622	3.6 (1.1)	3.4 (2.5; 5.0)
TG	2nd	297	2.1 (0.9)	1.9 (1.2; 3.3)	614	1.7 (0.6)	1.6 (1.1; 2.5)
	3rd	299	2.4 (0.8)	2.2 (1.5; 3.3)	622	2.7 (0.9)	2.5 (1.7; 3.9)
Glucose	2nd	302	4.4 (0.9)	4.2 (3.5; 5.8)	621	4.2 (0.5)	4.2 (3.8; 4.6)
	3rd	296	4.8 (1.1)	4.6 (3.6; 6.1)	578	4.2 (0.7)	4.1 3.6; 4.9)
Fasting state							
TG	2nd	62	2.0 (0.8)	1.8 (1.3; 3.0)	504	1.7 (0.6)	1.6 (1.1; 2.5)
	3rd	63	2.4 (0.8)	2.2 (1.5; 3.3)	511	2.6 (0.9)	2.5 (1.6; 3.9)
Glucose	2nd	63	4.1 (0.8)	3.9 (3.3; 5.0)	510	4.2 (0.4)	4.2 (3.8; 4.6)
	3rd	61	4.6 (1.0)	4.6 (3.5; 5.2)	471	4.1 (0.5)	4.0 (3.6; 4.7)

Supplementary Table 3. Comparison of the metabolic biomarkers across the trimesters for each sample with gestational ages ranging from the 22nd-28th and 33rd -39th week

		**Crude**			**Adjusted**[1]		
Sample	Biomarker	b	se	p	b	se	p
Rwanda	TC	0.76	0.07	<.0001	0.81	0.07	<.0001
	HDL	0.02	0.02	0.3937	0.03	0.02	0.1654
	LDL	0.59	0.07	<.0001	0.63	0.07	<.0001
	non-HDL	0.76	0.06	<.0001	0.80	0.06	<.0001
	TG	0.17	0.10	0.1005	0.18	0.11	0.1010
	Glucose	0.27	0.14	0.0488	0.23	0.15	0.1261
	Fasting state						
	TG	0.14	0.21	0.5035	0.20	0.23	0.3853
	Glucose	0.48	0.30	0.1321	0.55	0.33	0.1194
Leipzig	TC	0.69	0.03	<.0001	0.70	0.03	<.0001
	HDL	-0.16	0.01	<.0001	-0.16	0.01	<.0001
	LDL	0.57	0.03	<.0001	0.57	0.03	<.0001
	non-HDL	0.85	0.03	<.0001	0.85	0.03	<.0001
	TG	0.97	0.03	<.0001	0.97	0.03	<.0001
	Glucose	-0.03	0.03	0.2276	-0.03	0.03	0.2535
	Fasting state						
	TG	0.92	0.03	<.0001	0.92	0.03	<.0001
	Glucose	-0.12	0.02	<.0001	-0.12	0.02	<.0001

Note. b: mean difference comparing 3rd to 2nd trimester, se: standard error, TC: Total cholesterol, HDL: High-density lipoprotein cholesterol, LDL: Low-density lipoprotein, TG: Triglycerides.

[1] Adjusted for gestational age (2nd trimester), time between repeated measurements, maternal age, mid-upper arm circumference (2nd trimester), gravidity.

Supplementary Table 4. Comparison of the metabolic biomarkers across both samples with gestational ages ranging from the 22nd-28th and 33rd-39th week for each trimester

		Crude			Adjusted[1]		
Trimester	Biomarker	b	std	p	b	std	p
2nd	TC	1.75	0.13	<.0001	1.62	0.14	<.0001
	HDL	0.72	0.05	<.0001	0.71	0.06	<.0001
	LDL	1.73	0.12	<.0001	1.65	0.14	<.0001
	non-HDL	1.05	0.12	<.0001	0.93	0.14	<.0001
	TG	-0.58	0.08	<.0001	-0.63	0.09	<.0001
	Glucose	-0.17	0.06	0.0070	-0.28	0.07	<.0001
	Fasting state						
	TG	-0.47	0.15	0.0018	-0.54	0.16	0.0009
	Glucose	0.07	0.09	0.4724	0.01	0.09	0.9557
3rd	TC	1.79	0.11	<.0001	1.95	0.18	<.0001
	HDL	0.60	0.04	<.0001	0.60	0.06	<.0001
	LDL	1.73	0.11	<.0001	1.97	0.17	<.0001
	non-HDL	1.19	0.12	<.0001	1.36	0.18	<.0001
	TG	0.20	0.09	0.0254	0.23	0.14	0.1078
	Glucose	-0.46	0.07	<.0001	-0.43	0.13	0.0009
	Fasting state						
	TG	0.29	0.16	0.0776	0.28	0.25	0.2573
	Glucose	-0.60	0.10	<.0001	-0.66	0.17	0.0002

Note. b: mean difference comparing 3rd to 2nd trimester, se: standard error, TC: Total cholesterol, HDL: High-density lipoprotein cholesterol, LDL: Low-density lipoprotein, TG: Triglycerides.

[1] Adjusted for gestational age (2nd trimester), time between repeated measurements, maternal age, mid-upper arm circumference (2nd trimester), gravidity.

Supplementary Table 5. p-values for the interaction sample*trimester for each metabolic biomarker, in samples with gestational ages ranging from the 22nd-28th and 33rd-39th week

	**Crude**	**Adjusted**[1]
Biomarker	p-value	p-value
TC	0.4725	0.1557
HDL	<.0001	<.0001
LDL	0.7664	0.4519
non-HDL	0.2369	0.5652
TG	<.0001	<.0001
Glucose	0.0007	0.0025
Fasting state		
TG	<.0001	<.0001
Glucose	<.0001	<.0001

Note. TC: Total cholesterol, HDL: High-density lipoprotein cholesterol, LDL: Low-density lipoprotein, TG: Triglycerides.

[1] Adjusted for gestational age (2nd trimester), time between repeated measurements, maternal age, mid-upper arm circumference (2nd trimester), gravidity.

Supplementary Table 6. Distribution parameter and percentiles for the reference ranges of total cholesterol (TC)

			Distribution parameter				Percentiles						
Trimester	Sample	GA	mu	sigma	nu	tau	p05	p10	p25	p50	p75	p90	p95
2nd	Rwanda	16	3.90	0.21	0.35	2.28	2.71	2.94	3.36	3.90	4.50	5.05	5.39
	Rwanda	17	3.96	0.21	0.33	2.25	2.76	2.99	3.41	3.96	4.55	5.11	5.45
	Rwanda	18	4.01	0.21	0.31	2.22	2.81	3.04	3.46	4.01	4.61	5.17	5.52
	Rwanda	19	4.06	0.20	0.29	2.19	2.86	3.09	3.52	4.06	4.67	5.23	5.59
	Rwanda	20	4.12	0.20	0.27	2.16	2.91	3.14	3.57	4.12	4.72	5.30	5.66
	Rwanda	21	4.17	0.20	0.25	2.13	2.96	3.20	3.63	4.17	4.78	5.36	5.72
	Rwanda	22	4.23	0.20	0.23	2.10	3.01	3.25	3.68	4.23	4.84	5.42	5.79
	Rwanda	23	4.29	0.20	0.22	2.07	3.07	3.30	3.74	4.29	4.90	5.49	5.86
	Leipzig	23	6.00	0.18	-0.08	2.07	4.51	4.80	5.33	6.00	6.78	7.55	8.05
	Rwanda	24	4.35	0.20	0.20	2.04	3.12	3.36	3.80	4.35	4.95	5.55	5.93
	Leipzig	24	6.07	0.18	-0.06	2.04	4.55	4.84	5.38	6.07	6.84	7.62	8.13
	Rwanda	25	4.41	0.19	0.18	2.02	3.17	3.42	3.86	4.41	5.01	5.62	6.01
	Leipzig	25	6.13	0.18	-0.04	2.02	4.59	4.89	5.44	6.13	6.91	7.70	8.21
	Rwanda	26	4.46	0.19	0.16	1.99	3.23	3.47	3.92	4.46	5.08	5.68	6.08
	Leipzig	26	6.19	0.18	-0.02	1.99	4.63	4.94	5.50	6.19	6.98	7.77	8.29
	Rwanda	27	4.53	0.19	0.14	1.96	3.28	3.53	3.98	4.53	5.14	5.75	6.15
	Leipzig	27	6.26	0.18	0.00	1.96	4.67	4.99	5.56	6.26	7.05	7.85	8.37
3rd	Rwanda	28	4.59	0.19	0.12	1.93	3.34	3.59	4.04	4.59	5.20	5.82	6.23
	Leipzig	28	6.32	0.18	0.02	1.93	4.72	5.04	5.61	6.32	7.11	7.92	8.46
	Rwanda	29	4.65	0.19	0.10	1.91	3.40	3.65	4.10	4.65	5.26	5.89	6.30
	Leipzig	29	6.39	0.18	0.04	1.91	4.76	5.09	5.67	6.39	7.18	8.00	8.54

Rwanda	30	4.71	0.19	0.08	1.88	3.45	3.71	4.16	4.71	5.33	5.96	6.38
Leipzig	30	6.45	0.18	0.06	1.88	4.80	5.14	5.73	6.45	7.25	8.08	8.63
Rwanda	31	4.78	0.18	0.06	1.86	3.51	3.77	4.23	4.78	5.39	6.03	6.46
Leipzig	31	6.52	0.18	0.08	1.86	4.84	5.19	5.79	6.52	7.33	8.16	8.71
Rwanda	32	4.84	0.18	0.04	1.83	3.57	3.83	4.29	4.84	5.46	6.10	6.53
Leipzig	32	6.59	0.18	0.11	1.83	4.89	5.24	5.86	6.59	7.40	8.24	8.80
Rwanda	33	4.91	0.18	0.03	1.81	3.63	3.89	4.36	4.91	5.52	6.17	6.61
Leipzig	33	6.65	0.18	0.13	1.81	4.93	5.29	5.92	6.65	7.47	8.32	8.89
Rwanda	34	4.97	0.18	0.01	1.78	3.69	3.96	4.42	4.97	5.59	6.25	6.69
Leipzig	34	6.72	0.18	0.15	1.78	4.97	5.34	5.98	6.72	7.54	8.40	8.97
Rwanda	35	5.04	0.18	-0.01	1.76	3.76	4.02	4.49	5.04	5.66	6.32	6.77
Leipzig	35	6.79	0.18	0.17	1.76	5.02	5.39	6.04	6.79	7.62	8.48	9.06
Rwanda	36	5.11	0.18	-0.03	1.73	3.82	4.09	4.56	5.11	5.73	6.39	6.85
Leipzig	36	6.86	0.18	0.19	1.73	5.06	5.45	6.11	6.86	7.69	8.57	9.15
Rwanda	37	5.18	0.18	-0.05	1.71	3.88	4.15	4.63	5.18	5.80	6.47	6.94
Leipzig	37	6.93	0.18	0.21	1.71	5.11	5.50	6.17	6.93	7.77	8.65	9.24
Rwanda	38	5.25	0.17	-0.07	1.69	3.95	4.22	4.70	5.25	5.87	6.55	7.02
Rwanda	39	5.32	0.17	-0.09	1.66	4.01	4.29	4.77	5.32	5.94	6.62	7.10

Notes. The numbers displayed for gestational age (GA) represent the respective pregnancy week. The given pregnancy weeks differ between both samples for both trimesters.

Supplementary Table 7. Distribution parameter and percentiles for the reference ranges of high-density lipoprotein cholesterol (HDL)

			Distribution parameter				Percentiles						
Trimester	Sample	GA	mu	sigma	nu	tau	p05	p10	p25	p50	p75	p90	p95
2nd	Rwanda	16	1.39	0.22	0.46	2.34	0.94	1.03	1.18	1.39	1.61	1.81	1.93
	Rwanda	17	1.39	0.22	0.46	2.30	0.94	1.03	1.19	1.39	1.61	1.81	1.93
	Rwanda	18	1.39	0.22	0.46	2.27	0.94	1.03	1.19	1.39	1.61	1.81	1.94
	Rwanda	19	1.39	0.22	0.45	2.24	0.94	1.03	1.19	1.39	1.61	1.81	1.94
	Rwanda	20	1.39	0.22	0.45	2.21	0.94	1.02	1.19	1.39	1.61	1.82	1.94
	Rwanda	21	1.39	0.22	0.45	2.18	0.93	1.02	1.19	1.39	1.61	1.82	1.95
	Rwanda	22	1.39	0.22	0.45	2.15	0.93	1.02	1.19	1.39	1.61	1.82	1.95
	Rwanda	23	1.39	0.22	0.45	2.12	0.93	1.02	1.19	1.39	1.61	1.82	1.95
	Leipzig	23	2.15	0.21	0.30	2.12	1.49	1.62	1.85	2.15	2.48	2.80	3.00
	Rwanda	24	1.39	0.22	0.45	2.09	0.93	1.02	1.19	1.39	1.61	1.82	1.95
	Leipzig	24	2.14	0.21	0.30	2.09	1.47	1.60	1.84	2.14	2.47	2.78	2.99
	Rwanda	25	1.39	0.23	0.45	2.06	0.93	1.02	1.19	1.39	1.61	1.83	1.96
	Leipzig	25	2.12	0.22	0.30	2.06	1.46	1.59	1.82	2.12	2.45	2.77	2.97
	Rwanda	26	1.39	0.23	0.45	2.03	0.93	1.02	1.19	1.39	1.61	1.83	1.96
	Leipzig	26	2.11	0.22	0.30	2.03	1.44	1.57	1.81	2.11	2.43	2.76	2.96
	Rwanda	27	1.39	0.23	0.45	2.00	0.92	1.02	1.19	1.39	1.61	1.83	1.96
	Leipzig	27	2.09	0.22	0.30	2.00	1.42	1.56	1.80	2.09	2.42	2.74	2.95
3rd	Rwanda	28	1.39	0.23	0.45	1.97	0.92	1.02	1.19	1.39	1.61	1.83	1.97
	Leipzig	28	2.08	0.22	0.30	1.97	1.41	1.54	1.78	2.08	2.40	2.73	2.94
	Rwanda	29	1.39	0.23	0.45	1.94	0.92	1.02	1.19	1.39	1.61	1.83	1.97
	Leipzig	29	2.06	0.22	0.30	1.94	1.39	1.53	1.77	2.06	2.39	2.72	2.93

Rwanda	30	1.39	0.23	0.45	1.92	0.92	1.02	1.19	1.39	1.61	1.83	1.97
Leipzig	30	2.05	0.23	0.30	1.92	1.38	1.51	1.76	2.05	2.37	2.70	2.92
Rwanda	31	1.39	0.23	0.45	1.89	0.92	1.02	1.19	1.39	1.61	1.84	1.98
Leipzig	31	2.03	0.23	0.30	1.89	1.36	1.50	1.74	2.03	2.36	2.69	2.91
Rwanda	32	1.39	0.23	0.45	1.86	0.92	1.02	1.19	1.39	1.61	1.84	1.98
Leipzig	32	2.02	0.23	0.30	1.86	1.35	1.49	1.73	2.02	2.34	2.68	2.90
Rwanda	33	1.39	0.23	0.45	1.84	0.91	1.01	1.19	1.39	1.62	1.84	1.98
Leipzig	33	2.01	0.23	0.30	1.84	1.33	1.47	1.72	2.01	2.33	2.66	2.89
Rwanda	34	1.39	0.23	0.45	1.81	0.91	1.01	1.19	1.39	1.62	1.84	1.99
Leipzig	34	1.99	0.24	0.30	1.81	1.32	1.46	1.70	1.99	2.32	2.65	2.87
Rwanda	35	1.39	0.23	0.45	1.79	0.91	1.01	1.19	1.39	1.62	1.84	1.99
Leipzig	35	1.98	0.24	0.30	1.79	1.30	1.44	1.69	1.98	2.30	2.64	2.86
Rwanda	36	1.39	0.24	0.45	1.76	0.91	1.01	1.19	1.39	1.62	1.84	1.99
Leipzig	36	1.97	0.24	0.30	1.76	1.29	1.43	1.68	1.97	2.29	2.63	2.85
Rwanda	37	1.39	0.24	0.45	1.74	0.91	1.01	1.19	1.39	1.62	1.85	2.00
Leipzig	37	1.95	0.24	0.31	1.74	1.28	1.42	1.66	1.95	2.27	2.61	2.84
Rwanda	38	1.39	0.24	0.45	1.71	0.91	1.01	1.19	1.39	1.62	1.85	2.00
Rwanda	39	1.40	0.24	0.45	1.69	0.90	1.01	1.19	1.40	1.62	1.85	2.00

Notes. The numbers displayed for gestational age (GA) represent the respective pregnancy week. The given pregnancy weeks differ between both samples for both trimesters.

Supplementary Table 8. Distribution parameter and percentiles for the reference ranges of low-density lipoprotein (LDL)

			Distribution parameter				Percentiles						
Trimester	Sample	GA	mu	sigma	nu	tau	p05	p10	p25	p50	p75	p90	p95
2nd	Rwanda	16	1.68	0.45	1.27	1.97	0.53	0.78	1.23	1.72	2.19	2.59	2.83
	Rwanda	17	1.71	0.44	1.22	1.95	0.54	0.80	1.25	1.74	2.22	2.64	2.89
	Rwanda	18	1.75	0.43	1.18	1.94	0.56	0.82	1.28	1.78	2.26	2.69	2.95
	Rwanda	19	1.79	0.43	1.13	1.92	0.59	0.85	1.31	1.81	2.30	2.75	3.01
	Rwanda	20	1.83	0.42	1.08	1.91	0.61	0.88	1.34	1.84	2.34	2.80	3.07
	Rwanda	21	1.87	0.42	1.04	1.90	0.64	0.91	1.37	1.88	2.39	2.85	3.14
	Rwanda	22	1.91	0.41	0.99	1.88	0.68	0.94	1.40	1.91	2.43	2.91	3.20
	Rwanda	23	1.95	0.40	0.94	1.87	0.72	0.98	1.44	1.95	2.47	2.97	3.27
	Leipzig	23	3.58	0.27	0.25	1.87	2.22	2.49	2.97	3.58	4.26	4.99	5.47
	Rwanda	24	1.99	0.40	0.90	1.86	0.76	1.02	1.48	1.99	2.52	3.02	3.34
	Leipzig	24	3.62	0.27	0.25	1.86	2.25	2.52	3.02	3.62	4.32	5.05	5.55
	Rwanda	25	2.03	0.39	0.85	1.84	0.81	1.07	1.52	2.03	2.57	3.08	3.41
	Leipzig	25	3.67	0.27	0.26	1.84	2.28	2.55	3.06	3.67	4.38	5.12	5.62
	Rwanda	26	2.08	0.39	0.80	1.83	0.85	1.11	1.57	2.08	2.62	3.14	3.48
	Leipzig	26	3.72	0.27	0.27	1.83	2.30	2.59	3.10	3.72	4.43	5.19	5.69
	Rwanda	27	2.12	0.38	0.76	1.82	0.91	1.16	1.61	2.12	2.67	3.21	3.55
	Leipzig	27	3.77	0.27	0.27	1.82	2.33	2.62	3.14	3.77	4.49	5.25	5.77
3rd	Rwanda	28	2.17	0.38	0.71	1.80	0.96	1.21	1.66	2.17	2.72	3.27	3.62
	Leipzig	28	3.82	0.27	0.28	1.80	2.36	2.65	3.18	3.82	4.55	5.32	5.85
	Rwanda	29	2.21	0.37	0.66	1.79	1.01	1.26	1.70	2.21	2.77	3.33	3.70
	Leipzig	29	3.87	0.27	0.28	1.79	2.39	2.69	3.23	3.87	4.61	5.39	5.92

Rwanda	30	2.26	0.37	0.62	1.78	1.06	1.31	1.75	2.26	2.82	3.40	3.78
Leipzig	30	3.93	0.27	0.29	1.78	2.42	2.72	3.27	3.93	4.67	5.46	6.00
Rwanda	31	2.31	0.36	0.57	1.77	1.12	1.36	1.80	2.31	2.88	3.46	3.86
Leipzig	31	3.98	0.27	0.30	1.77	2.45	2.76	3.32	3.98	4.73	5.53	6.08
Rwanda	32	2.36	0.36	0.52	1.75	1.17	1.41	1.85	2.36	2.93	3.53	3.94
Leipzig	32	4.03	0.27	0.30	1.75	2.48	2.80	3.36	4.03	4.79	5.61	6.16
Rwanda	33	2.41	0.35	0.48	1.74	1.23	1.47	1.90	2.41	2.99	3.60	4.02
Leipzig	33	4.09	0.27	0.31	1.74	2.51	2.83	3.41	4.09	4.85	5.68	6.24
Rwanda	34	2.46	0.35	0.43	1.73	1.28	1.52	1.95	2.46	3.04	3.67	4.10
Leipzig	34	4.14	0.27	0.32	1.73	2.54	2.87	3.46	4.14	4.92	5.75	6.33
Rwanda	35	2.52	0.34	0.38	1.72	1.34	1.57	2.00	2.52	3.10	3.74	4.19
Leipzig	35	4.20	0.27	0.32	1.72	2.57	2.91	3.50	4.20	4.98	5.83	6.41
Rwanda	36	2.57	0.34	0.34	1.70	1.39	1.63	2.06	2.57	3.16	3.82	4.27
Leipzig	36	4.25	0.27	0.33	1.70	2.60	2.95	3.55	4.25	5.05	5.91	6.50
Rwanda	37	2.62	0.33	0.29	1.69	1.45	1.68	2.11	2.62	3.22	3.89	4.36
Leipzig	37	4.31	0.28	0.34	1.69	2.63	2.98	3.60	4.31	5.11	5.98	6.58
Rwanda	38	2.68	0.33	0.24	1.68	1.50	1.74	2.17	2.68	3.28	3.96	4.45
Rwanda	39	2.74	0.32	0.20	1.67	1.56	1.80	2.22	2.74	3.34	4.04	4.54

Notes. The numbers displayed for gestational age (GA) represent the respective pregnancy week. The given pregnancy weeks differ between both samples for both trimesters.

Supplementary Table 9. Distribution parameter and percentiles for the reference ranges of non-high-density lipoprotein cholesterol (non-HDL)

			Distribution parameter				Percentiles						
Trimester	Sample	GA	mu	sigma	nu	tau	p05	p10	p25	p50	p75	p90	p95
2nd	Rwanda	16	2.51	0.30	0.61	2.48	1.40	1.60	1.99	2.51	3.08	3.58	3.87
	Rwanda	17	2.56	0.30	0.59	2.43	1.44	1.65	2.04	2.56	3.13	3.64	3.93
	Rwanda	18	2.61	0.30	0.57	2.38	1.48	1.69	2.09	2.61	3.18	3.70	4.00
	Rwanda	19	2.66	0.29	0.55	2.34	1.53	1.74	2.14	2.66	3.24	3.76	4.07
	Rwanda	20	2.71	0.29	0.52	2.29	1.57	1.78	2.19	2.71	3.29	3.82	4.14
	Rwanda	21	2.76	0.29	0.50	2.25	1.62	1.83	2.24	2.76	3.35	3.89	4.22
	Rwanda	22	2.82	0.28	0.48	2.20	1.66	1.88	2.29	2.82	3.40	3.95	4.29
	Rwanda	23	2.87	0.28	0.46	2.16	1.71	1.93	2.34	2.87	3.46	4.02	4.37
	Leipzig	23	3.81	0.27	0.18	2.16	2.40	2.65	3.15	3.81	4.58	5.36	5.85
	Rwanda	24	2.93	0.28	0.44	2.12	1.76	1.98	2.40	2.93	3.52	4.09	4.44
	Leipzig	24	3.88	0.27	0.19	2.12	2.44	2.71	3.21	3.88	4.66	5.44	5.95
	Rwanda	25	2.98	0.28	0.42	2.08	1.80	2.03	2.45	2.98	3.58	4.15	4.52
	Leipzig	25	3.96	0.27	0.21	2.08	2.48	2.76	3.28	3.96	4.74	5.53	6.04
	Rwanda	26	3.04	0.27	0.40	2.04	1.85	2.08	2.51	3.04	3.64	4.22	4.60
	Leipzig	26	4.03	0.27	0.22	2.04	2.53	2.81	3.34	4.03	4.82	5.62	6.14
	Rwanda	27	3.10	0.27	0.38	2.00	1.90	2.14	2.56	3.10	3.70	4.30	4.68
	Leipzig	27	4.10	0.27	0.24	2.00	2.58	2.87	3.41	4.10	4.90	5.71	6.24
3rd	Rwanda	28	3.16	0.27	0.35	1.96	1.95	2.19	2.62	3.16	3.76	4.37	4.76
	Leipzig	28	4.18	0.27	0.25	1.96	2.62	2.92	3.48	4.18	4.98	5.80	6.34
	Rwanda	29	3.22	0.27	0.33	1.92	2.01	2.25	2.68	3.22	3.82	4.44	4.84
	Leipzig	29	4.26	0.27	0.27	1.92	2.67	2.98	3.55	4.26	5.06	5.90	6.45

Rwanda	30	3.28	0.26	0.31	1.89	2.06	2.30	2.74	3.28	3.89	4.51	4.93
Leipzig	30	4.34	0.27	0.28	1.89	2.72	3.04	3.62	4.34	5.15	5.99	6.55
Rwanda	31	3.34	0.26	0.29	1.85	2.11	2.36	2.81	3.34	3.95	4.59	5.01
Leipzig	31	4.42	0.26	0.30	1.85	2.77	3.10	3.69	4.42	5.24	6.09	6.66
Rwanda	32	3.41	0.26	0.27	1.81	2.17	2.42	2.87	3.41	4.02	4.67	5.10
Leipzig	32	4.50	0.26	0.31	1.81	2.82	3.16	3.77	4.50	5.32	6.19	6.77
Rwanda	33	3.47	0.26	0.25	1.78	2.23	2.48	2.93	3.47	4.09	4.74	5.19
Leipzig	33	4.58	0.26	0.33	1.78	2.87	3.22	3.85	4.58	5.41	6.29	6.88
Rwanda	34	3.54	0.25	0.23	1.75	2.28	2.54	3.00	3.54	4.16	4.82	5.28
Leipzig	34	4.67	0.26	0.34	1.75	2.92	3.28	3.92	4.67	5.50	6.39	6.99
Rwanda	35	3.61	0.25	0.21	1.71	2.34	2.60	3.07	3.61	4.23	4.90	5.37
Leipzig	35	4.76	0.26	0.36	1.71	2.98	3.35	4.00	4.76	5.60	6.50	7.11
Rwanda	36	3.68	0.25	0.18	1.68	2.40	2.67	3.13	3.68	4.30	4.98	5.46
Leipzig	36	4.85	0.26	0.37	1.68	3.03	3.42	4.08	4.85	5.69	6.60	7.22
Rwanda	37	3.75	0.25	0.16	1.65	2.46	2.73	3.20	3.75	4.37	5.06	5.56
Leipzig	37	4.94	0.26	0.39	1.65	3.09	3.48	4.17	4.94	5.79	6.71	7.34
Rwanda	38	3.82	0.24	0.14	1.61	2.52	2.80	3.28	3.82	4.44	5.15	5.65
Rwanda	39	3.89	0.24	0.12	1.58	2.59	2.87	3.35	3.89	4.52	5.23	5.75

Notes. The numbers displayed for gestational age (GA) represent the respective pregnancy week. The given pregnancy weeks differ between both samples for both trimesters.

Supplementary Table 10. Distribution parameter and percentiles for the reference ranges of triglyceride (TG)

			Distribution parameter				Percentiles						
Trimester	Sample	GA	mu	sigma	nu	tau	p05	p10	p25	p50	p75	p90	p95
2nd	Rwanda	16	1.74	0.39	-0.65	2.67	1.03	1.12	1.34	1.74	2.37	3.21	3.89
	Rwanda	17	1.77	0.38	-0.64	2.60	1.05	1.15	1.37	1.77	2.39	3.20	3.87
	Rwanda	18	1.80	0.37	-0.63	2.54	1.08	1.18	1.40	1.80	2.41	3.20	3.85
	Rwanda	19	1.83	0.36	-0.61	2.47	1.10	1.20	1.43	1.83	2.43	3.21	3.83
	Rwanda	20	1.86	0.36	-0.60	2.41	1.12	1.23	1.47	1.86	2.45	3.21	3.82
	Rwanda	21	1.89	0.35	-0.59	2.35	1.15	1.26	1.50	1.89	2.47	3.22	3.81
	Rwanda	22	1.92	0.35	-0.57	2.29	1.18	1.29	1.53	1.92	2.49	3.22	3.81
	Rwanda	23	1.95	0.34	-0.56	2.23	1.20	1.32	1.56	1.95	2.52	3.23	3.80
	Leipzig	23	1.52	0.31	-0.38	2.23	0.95	1.04	1.23	1.52	1.91	2.36	2.68
	Rwanda	24	1.98	0.33	-0.55	2.18	1.23	1.35	1.60	1.98	2.54	3.24	3.80
	Leipzig	24	1.58	0.31	-0.35	2.18	0.98	1.08	1.28	1.58	1.98	2.45	2.79
	Rwanda	25	2.02	0.33	-0.53	2.12	1.26	1.38	1.63	2.02	2.56	3.25	3.80
	Leipzig	25	1.65	0.32	-0.32	2.12	1.02	1.12	1.33	1.65	2.06	2.55	2.91
	Rwanda	26	2.05	0.32	-0.52	2.07	1.29	1.41	1.67	2.05	2.59	3.27	3.81
	Leipzig	26	1.71	0.32	-0.30	2.07	1.05	1.17	1.39	1.71	2.15	2.65	3.03
	Rwanda	27	2.09	0.32	-0.51	2.02	1.31	1.44	1.70	2.09	2.61	3.28	3.81
	Leipzig	27	1.79	0.32	-0.27	2.02	1.09	1.21	1.45	1.79	2.23	2.76	3.15
3rd	Rwanda	28	2.12	0.31	-0.49	1.96	1.34	1.48	1.74	2.12	2.64	3.30	3.82
	Leipzig	28	1.86	0.32	-0.24	1.96	1.13	1.26	1.51	1.86	2.32	2.87	3.28
	Rwanda	29	2.16	0.30	-0.48	1.91	1.37	1.51	1.78	2.16	2.67	3.31	3.83
	Leipzig	29	1.94	0.32	-0.22	1.91	1.17	1.30	1.57	1.94	2.41	2.99	3.41

Rwanda	30	2.19	0.30	-0.47	1.87	1.41	1.54	1.81	2.19	2.70	3.33	3.84
Leipzig	30	2.02	0.33	-0.19	1.87	1.21	1.35	1.63	2.02	2.51	3.11	3.55
Rwanda	31	2.23	0.29	-0.45	1.82	1.44	1.58	1.85	2.23	2.72	3.35	3.85
Leipzig	31	2.10	0.33	-0.16	1.82	1.25	1.40	1.70	2.10	2.61	3.23	3.70
Rwanda	32	2.26	0.29	-0.44	1.77	1.47	1.61	1.89	2.26	2.75	3.37	3.87
Leipzig	32	2.19	0.33	-0.14	1.77	1.29	1.46	1.77	2.19	2.72	3.36	3.85
Rwanda	33	2.30	0.28	-0.43	1.73	1.50	1.65	1.93	2.30	2.78	3.40	3.88
Leipzig	33	2.28	0.33	-0.11	1.73	1.34	1.51	1.84	2.28	2.83	3.50	4.01
Rwanda	34	2.34	0.28	-0.41	1.69	1.54	1.69	1.97	2.34	2.81	3.42	3.90
Leipzig	34	2.37	0.33	-0.08	1.69	1.38	1.57	1.92	2.37	2.94	3.64	4.17
Rwanda	35	2.38	0.27	-0.40	1.64	1.57	1.73	2.01	2.38	2.84	3.44	3.92
Leipzig	35	2.47	0.34	-0.05	1.64	1.43	1.63	2.00	2.47	3.06	3.78	4.34
Rwanda	36	2.42	0.27	-0.39	1.60	1.61	1.76	2.06	2.42	2.88	3.47	3.94
Leipzig	36	2.57	0.34	-0.03	1.60	1.48	1.69	2.08	2.57	3.18	3.93	4.52
Rwanda	37	2.46	0.26	-0.37	1.56	1.64	1.80	2.10	2.46	2.91	3.49	3.96
Leipzig	37	2.68	0.34	0.00	1.56	1.53	1.75	2.17	2.68	3.30	4.09	4.70
Rwanda	38	2.50	0.26	-0.36	1.52	1.68	1.84	2.14	2.50	2.94	3.52	3.98
Rwanda	39	2.54	0.25	-0.35	1.48	1.72	1.89	2.19	2.54	2.98	3.55	4.01

Notes. The numbers displayed for gestational age (GA) represent the respective pregnancy week. The given pregnancy weeks differ between both samples for both trimesters.

Supplementary Table 11. Distribution parameter and percentiles for the reference ranges of glucose

			**Distribution parameter**					**Percentiles**						
Trimester	Sample	GA	mu	sigma	nu	tau	p05	p10	p25	p50	p75	p90	p95
2nd	Rwanda	16	4.19	0.18	-1.38	1.84	3.27	3.44	3.76	4.19	4.77	5.52	6.14
	Rwanda	17	4.21	0.18	-1.32	1.78	3.27	3.44	3.77	4.21	4.79	5.55	6.18
	Rwanda	18	4.23	0.18	-1.26	1.73	3.27	3.45	3.79	4.23	4.81	5.58	6.22
	Rwanda	19	4.25	0.19	-1.21	1.68	3.27	3.46	3.80	4.25	4.83	5.61	6.26
	Rwanda	20	4.27	0.19	-1.15	1.64	3.27	3.46	3.82	4.27	4.86	5.64	6.30
	Rwanda	21	4.29	0.19	-1.09	1.59	3.27	3.47	3.83	4.29	4.88	5.67	6.34
	Rwanda	22	4.31	0.19	-1.03	1.54	3.26	3.47	3.85	4.31	4.90	5.70	6.37
	Rwanda	23	4.33	0.20	-0.98	1.50	3.26	3.48	3.87	4.33	4.92	5.72	6.41
	Leipzig	23	4.18	0.08	-2.10	1.50	3.71	3.81	3.99	4.18	4.40	4.68	4.90
	Rwanda	24	4.35	0.20	-0.92	1.46	3.26	3.48	3.88	4.35	4.94	5.75	6.45
	Leipzig	24	4.17	0.09	-1.97	1.46	3.68	3.79	3.97	4.17	4.40	4.69	4.92
	Rwanda	25	4.37	0.20	-0.86	1.42	3.26	3.49	3.90	4.37	4.96	5.78	6.49
	Leipzig	25	4.16	0.09	-1.84	1.42	3.65	3.77	3.96	4.16	4.40	4.70	4.94
	Rwanda	26	4.39	0.21	-0.80	1.38	3.25	3.49	3.92	4.39	4.98	5.81	6.52
	Leipzig	26	4.15	0.09	-1.71	1.38	3.62	3.74	3.94	4.15	4.40	4.71	4.97
	Rwanda	27	4.41	0.21	-0.75	1.34	3.25	3.50	3.93	4.41	5.00	5.83	6.56
	Leipzig	27	4.15	0.10	-1.58	1.34	3.59	3.72	3.93	4.15	4.40	4.73	4.99
3rd	Rwanda	28	4.43	0.21	-0.69	1.30	3.24	3.50	3.95	4.43	5.02	5.86	6.59
	Leipzig	28	4.14	0.10	-1.45	1.30	3.56	3.70	3.91	4.14	4.40	4.74	5.02
	Rwanda	29	4.45	0.21	-0.63	1.26	3.24	3.51	3.97	4.45	5.04	5.89	6.63
	Leipzig	29	4.13	0.11	-1.32	1.26	3.53	3.67	3.90	4.13	4.39	4.75	5.04

Rwanda	30	4.47	0.22	-0.57	1.23	3.23	3.51	3.98	4.47	5.06	5.91	6.66
Leipzig	30	4.12	0.11	-1.19	1.23	3.49	3.64	3.88	4.12	4.39	4.76	5.07
Rwanda	31	4.49	0.22	-0.51	1.19	3.23	3.52	4.00	4.49	5.08	5.94	6.69
Leipzig	31	4.11	0.12	-1.06	1.19	3.45	3.62	3.87	4.11	4.39	4.78	5.10
Rwanda	32	4.51	0.22	-0.46	1.16	3.22	3.52	4.02	4.51	5.10	5.97	6.73
Leipzig	32	4.11	0.12	-0.93	1.16	3.42	3.59	3.85	4.11	4.39	4.79	5.12
Rwanda	33	4.53	0.22	-0.40	1.12	3.21	3.53	4.04	4.53	5.12	5.99	6.76
Leipzig	33	4.10	0.13	-0.80	1.12	3.38	3.56	3.84	4.10	4.39	4.80	5.15
Rwanda	34	4.56	0.23	-0.34	1.09	3.21	3.53	4.06	4.56	5.14	6.01	6.79
Leipzig	34	4.09	0.13	-0.66	1.09	3.33	3.53	3.82	4.09	4.39	4.82	5.18
Rwanda	35	4.58	0.23	-0.28	1.06	3.20	3.54	4.08	4.58	5.16	6.04	6.82
Leipzig	35	4.08	0.14	-0.53	1.06	3.29	3.50	3.81	4.08	4.39	4.83	5.20
Rwanda	36	4.60	0.23	-0.23	1.03	3.19	3.54	4.10	4.60	5.17	6.06	6.85
Leipzig	36	4.07	0.15	-0.40	1.03	3.25	3.46	3.79	4.07	4.38	4.84	5.23
Rwanda	37	4.62	0.24	-0.17	1.00	3.18	3.55	4.12	4.62	5.19	6.09	6.88
Leipzig	37	4.06	0.15	-0.27	1.00	3.20	3.43	3.77	4.06	4.38	4.85	5.25
Rwanda	38	4.64	0.24	-0.11	0.97	3.17	3.55	4.14	4.64	5.21	6.11	6.91
Rwanda	39	4.66	0.24	-0.05	0.94	3.16	3.56	4.16	4.66	5.23	6.13	6.94

Notes. The numbers displayed for gestational age (GA) represent the respective pregnancy week. The given pregnancy weeks differ between both samples for both trimesters.

Supplementary Table 12. *P*-values for the interaction between cohort and trimester for each metabolic biomarker

	***P*-value**	
	**Crude**	**Adjusted***
**TC**	0.0100	0.0112
**HDL**	<0.0001	<0.0001
**LDL**	0.1019	0.1051
**non-HDL**	0.4725	0.4508
**TG**	<0.0001	<0.0001
**Glucose**	<0.0001	<0.0001
**Fasting state**		
TG	<0.0001	<0.0001
Glucose	<0.0001	<0.0001

HDL – high-density lipoprotein cholesterol, LDL – low-density lipoprotein, TC – total cholesterol, TG – triglycerides

*Adjusted for gestational age (second trimester), time between repeated measurements, maternal age, mid-upper arm circumference (second trimester), and gravidity.

STROBE Statement—checklist of items that should be included in reports of observational studies – for the article, **Differences in Metabolic Adaptations during Mid and Late Pregnancy: A Comparative Cohort Study between Rwanda and Germany**

	Item No.	Recommendation	Page No.	Yes/No or N/A
**Title and abstract**	1	(*a*) Indicate the study's design with a commonly used term in the title or the abstract	1	yes
		(*b*) Provide in the abstract an informative and balanced summary of what was done and what was found	2-3	yes
**Introduction**				
Background/rationale	2	Explain the scientific background and rationale for the investigation being reported	5	yes
Objectives	3	State specific objectives, including any prespecified hypotheses	5-6	yes
**Methods**				
Study design	4	Present key elements of study design early in the paper	6-8	yes
Setting	5	Describe the setting, locations, and relevant dates, including periods of recruitment, exposure, follow-up, and data collection	6-8	
Participants	6	(*a*) *Cohort study*—Give the eligibility criteria, and the sources and methods of selection of participants. Describe methods of follow-up<br>*Case-control study*—Give the eligibility criteria, and the sources and methods of case ascertainment and control selection. Give the rationale for the choice of cases and controls<br>*Cross-sectional study*—Give the eligibility criteria, and the sources and methods of selection of participants	6-8	yes
		(*b*) *Cohort study*—For matched studies, give matching criteria and number of exposed and unexposed<br>*Case-control study*—For matched studies, give matching criteria and the number of controls per case		n/a
Variables	7	Clearly define all outcomes, exposures, predictors, potential confounders, and effect modifiers. Give diagnostic criteria, if applicable	6-8	yes
Data sources/ measurement	8	For each variable of interest, give sources of data and details of methods of assessment (measurement). Describe comparability of assessment methods if there is more than one group	6-8	yes
Bias	9	Describe any efforts to address potential sources of bias	17-18	yes
Study size	10	Explain how the study size was arrived at	6	yes

Continued on next page

	Item No.	Recommendation	Page No.	Yes/No or N/A
Quantitative variables	11	Explain how quantitative variables were handled in the analyses. If applicable, describe which groupings were chosen and why	6-8	yes
Statistical methods	12	(*a*) Describe all statistical methods, including those used to control for confounding	6-8	yes
		(*b*) Describe any methods used to examine subgroups and interactions		n/a
		(*c*) Explain how missing data were addressed		n/a
		(*d*) *Cohort study*—If applicable, explain how loss to follow-up was addressed<br>*Case-control study*—If applicable, explain how matching of cases and controls was addressed<br>*Cross-sectional study*—If applicable, describe analytical methods taking account of sampling strategy		n/a
		(*e*) Describe any sensitivity analyses	7	yes
**Results**				
Participants	13	(a) Report numbers of individuals at each stage of study—eg numbers potentially eligible, examined for eligibility, confirmed eligible, included in the study, completing follow-up, and analysed	8-13 and supplement	yes
		(b) Give reasons for non-participation at each stage		n/a
		(c) Consider use of a flow diagram		n/a
Descriptive data	14	(a) Give characteristics of study participants (eg demographic, clinical, social) and information on exposures and potential confounders	8-13 and supplement	yes
		(b) Indicate number of participants with missing data for each variable of interest		n/a
		(c) *Cohort study*—Summarise follow-up time (eg, average and total amount)	8-13	yes
Outcome data	15	*Cohort study*—Report numbers of outcome events or summary measures over time	8-13 and supplement	yes
		Case-control study—Report numbers in each exposure category, or summary measures of exposure		n/a
		Cross-sectional study—Report numbers of outcome events or summary measures		n/a
Main results	16	(*a*) Give unadjusted estimates and, if applicable, confounder-adjusted estimates and their precision (eg, 95% confidence interval). Make clear which confounders were adjusted for and why they were included	8-13, 17-19 and supplement	yes
		(*b*) Report category boundaries when continuous variables were categorized	8-13 and supplement	yes
		(*c*) If relevant, consider translating estimates of relative risk into absolute risk for a meaningful time period		n/a

Continued on next page

	Item No.	Recommendation	Page No.	Yes/No or N/A
Other analyses	17	Report other analyses done—eg analyses of subgroups and interactions, and sensitivity analyses	8-13	yes
**Discussion**				
Key results	18	Summarise key results with reference to study objectives	13	yes
Limitations	19	Discuss limitations of the study, taking into account sources of potential bias or imprecision. Discuss both direction and magnitude of any potential bias	17-19	yes
Interpretation	20	Give a cautious overall interpretation of results considering objectives, limitations, multiplicity of analyses, results from similar studies, and other relevant evidence	13-19	yes
Generalisability	21	Discuss the generalisability (external validity) of the study results	13-19	yes
**Other information**				
Funding	22	Give the source of funding and the role of the funders for the present study and, if applicable, for the original study on which the present article is based	21	yes
